# Estimating the epidemiological and economic impact of providing nutritional care for tuberculosis-affected households across India: a modelling study

**DOI:** 10.1016/S2214-109X(24)00505-9

**Published:** 2025-01-14

**Authors:** Christopher Finn McQuaid, Rebecca A Clark, Richard G White, Roel Bakker, Peter Alexander, Roslyn Henry, Banurekha Velayutham, Malaisamy Muniyandi, Pranay Sinha, Madhavi Bhargava, Anurag Bhargava, Rein M G J Houben

**Affiliations:** a TB Modelling Group, TB Centre, and Centre for Mathematical Modelling of Infectious Diseases, Department of Infectious Disease Epidemiology, London School of Hygiene & Tropical Medicine, London, UK; b KNCV Tuberculosis Foundation, The Hague, Netherlands; c School of Geosciences, University of Edinburgh, Edinburgh, UK; d School of Biological Sciences, University of Aberdeen, King's College, Aberdeen, UK; e ICMR–National Institute for Research in Tuberculosis, Chennai, India; f Section of Infectious Diseases, Department of Medicine, Boston University Chobanian & Avedisian School of Medicine, Boston, MA, USA; g Department of Community Medicine, Yenepoya Medical College, Mangalore, India; h Center for Nutrition Studies, Yenepoya University, Mangalore, India; i Department of Medicine, Yenepoya Medical College Hospital, Mangalore, India; j Department of Medicine, McGill University, Montreal, QC, Canada

## Abstract

**Background:**

Approximately 20% of global tuberculosis incidence is attributable to undernutrition, increasing to more than a third in India. Targeting nutritional interventions to tuberculosis-affected households is a policy priority, but understanding of epidemiological and economic impacts is limited. We aimed to estimate the population-level epidemiological and economic effect of such an intervention.

**Methods:**

We used a previously published, age-stratified, compartmental transmission model of tuberculosis in India, and incorporated explicit BMI strata linked to disease progression and treatment outcomes. We used results from a recent trial of an intervention in which nutritional support in the form of food baskets was provided to people initiating tuberculosis treatment and to their household contacts (1200 kcal for patients and 750 kcal for contacts) to inform estimates of the impact and costs of nutritional support. We estimated the numbers of cases of tuberculosis disease and deaths due to tuberculosis disease that could be averted from 2023 to 2035 under the intervention scenario.

**Findings:**

Compared with a baseline with no nutritional intervention, at 50% coverage of adults on tuberculosis treatment and their households (around 23% of households affected by incident tuberculosis in India), providing the nutritional support intervention could prevent 361 200 (95% uncertainty interval 318 000–437 700) tuberculosis deaths and 880 700 (802 700–974 900) disease episodes from 2023 to 2035. This would be equivalent to averting approximately 4·6% (4·2–5·5) tuberculosis deaths and 2·2% (2·1–2·4) tuberculosis episodes. The additional health system cost would be US$1349 million (1221–1492), with an incremental cost-effectiveness ratio of $167 (147–187) per disability-adjusted life-year averted. The median number of households needed to treat to prevent one tuberculosis death was 24·4 and to prevent one tuberculosis case was 10·0.

**Interpretation:**

A nutritional intervention for tuberculosis-affected households could avert a substantial amount of tuberculosis disease and death in India, and would be highly likely to be cost-effective on the basis of the tuberculosis-specific benefits alone.

**Funding:**

None.

**Translations:**

For the Bangla and Hindi translations of the abstract see Supplementary Materials section.

## Introduction

Globally, tuberculosis disease remains one of the leading causes of mortality due to a single pathogen. Exposure to *Mycobacterium tuberculosis* is widespread in countries with a high tuberculosis burden; however, most individuals contain or clear their infection.^1^ Comorbidities such as HIV, diabetes, and undernutrition can impair the control of *M tuberculosis*, increasing the risk of progressing to disease^2^ and unfavourable outcomes.^3^ Undernutrition is the leading risk factor for tuberculosis globally, accounting for 20% of annual tuberculosis incidence, and is a leading comorbidity in patients with tuberculosis.^1^ In recent years, the number of people with food insecurity has increased globally, highlighting the urgent need to incorporate nutritional interventions into tuberculosis elimination efforts.

Ecological observations and evidence from well documented cohorts have supported the benefit of improved nutrition in the decline of tuberculosis incidence in countries that now have a low tuberculosis burden.^4^ Despite insufficient research on the effect of nutritional interventions on treatment outcomes,^5^ in 2013, WHO recommended nutritional assessment, counselling, and support in selected groups as integral components of tuberculosis care.^6^ Globally, programmatic implementation of this recommendation is sparse;^7^ however, in India, the tuberculosis elimination programme established a direct benefit transfer in 2018 to enable a nutritious diet.^8^ The recent RATIONS cluster-randomised controlled trial in the Indian state of Jharkhand—a setting with high prevalence of poverty, undernutrition, and tuberculosis—showed for the first time evidence of the effect of a macronutritional support intervention in reducing tuberculosis incidence in the household contacts of adult patients with tuberculosis,^9^ as well as improving treatment outcomes for the patients themselves.^10^

**Research in context**

**Evidence before this study**

We searched PubMed with the terms (“tuberculosis” OR “tubercul*”) AND (“nutrition*” OR “nutrient*” OR “diet*” OR “vitamin*”) AND (“household”) AND (“model*” OR “cost-effect*”) for articles published from database inception to May 30, 2024, in English. We found 55 studies, from which we identified two studies that had estimated the cost-effectiveness of nutritional supplementation. One modelling study estimated the potential impact of nutritional interventions, including at a household level. One cluster-randomised controlled trial estimated the number needed to treat to prevent tuberculosis incidence. A 2022 study by Sinha and colleagues used a Markov model from the provider perspective to estimate the effect of providing nutritional support to undernourished individuals in India, finding that a substantial burden of tuberculosis in this population could be averted through this likely cost-effective intervention. Focusing on undernutrition in household contacts alone had a lower incremental cost-effectiveness ratio (ICER) than the general population. In a 2024 study (preprint), Sinha and colleagues took a similar approach again in India but considered household contacts in general, and also found this intervention to be cost-effective at most willingness-to-pay thresholds. Mandal and colleagues used a transmission model to estimate the epidemiological effect of providing nutritional support to undernourished household contacts of patients with tuberculosis across the southeast Asian region, finding a sizeable proportion of tuberculosis could be averted. Meanwhile, Bhargava and colleagues (2023) showed in a trial of nutritional supplements in India that around 30 households needed to receive the intervention to prevent one case of incident tuberculosis in 2 years.

**Added value of this study**

Our study is the first to evaluate the impact and cost-effectiveness of a nutritional intervention for tuberculosis-affected households while considering the effect on onward transmission of reduced tuberculosis disease. We compared different recipients of the intervention (both individuals on tuberculosis treatment and their household contacts), providing national-level estimates of the health benefits, costs to both the health system and to wider society, and ICERs for India.

**Implications of all the available evidence**

We found that if India can deliver a nutritional intervention to 50% of households in which individuals are receiving tuberculosis treatment, nearly 900 000 episodes of tuberculosis would be averted, and nearly 400 000 deaths, at a cost of $167 per disability-adjusted life-year averted. Nutritional supplementation is likely to have substantial tuberculosis-related health benefits and be cost-effective at most willingness-to-pay thresholds, even at the national level. A focus on states with high levels of undernutrition is likely to avert a higher proportion of the tuberculosis burden, further improving the value for money. Considering the equity and health benefits beyond tuberculosis, our study likely underestimates both impact and cost-effectiveness.

In this study, we modelled the effect of scaling up such an intervention nationally in India to estimate the potential impact of the intervention on tuberculosis incidence and mortality in the longer term, including averted transmission, as well as the cost and cost-effectiveness of introducing nutritional support to those receiving tuberculosis treatment and their household contacts.

## Methods

### Model

We used a previously published, age-stratified, compartmental transmission model of tuberculosis in India.^11^ The methods are reproduced here with modifications described to simulate the intervention, and are visualised in appendix 3 (p 2). The model was coded in R version 4.3.3 and 4.3.2.

We incorporated explicit BMI strata into the model (<17·0 kg/m^2^, 17·0 kg/m^2^ to <18·5 kg/m^2^, 18·5 kg/m^2^ to <25·0 kg/m^2^, and ≥25·0 kg/m^2^). Trends in BMI were obtained from the UN Population Division (2019 revision),^12^ the Global Health Observatory,^13^ and India National Family Health Surveys,^14^ and future BMI projections were obtained from the LandSyMM food system model middle-of-the-road scenario.^15^ We used weight-for-height SDs for children younger than 5 years and BMI SDs for children and adolescents aged 5–19 years. We re-estimated the relative BMI-specific risk of progression and reversion to tuberculosis disease (similar to methods used elsewhere)^2^, ^16^ and treatment outcomes^3^ (appendix 3 p 8). The risk of *M tuberculosis* infection^17^ and time to diagnosis were assumed to be the same across BMI strata.

### Calibration and uncertainty

The model was fitted to 15 calibration targets to represent the tuberculosis epidemic in India: the tuberculosis incidence, mortality, and case notification rates (overall and by age) in the calendar years 2000 and 2020,^1^ the tuberculosis disease prevalence (overall and for adults) in 2015 and in 2021,^18^, ^19^ the overall prevalence of *M tuberculosis* infection (not including current tuberculosis disease) in 2021,^18^ and the fraction of asymptomatic tuberculosis among active tuberculosis disease.^20^ The effect of the COVID-19 pandemic was thus implicitly included (because the case notification and prevalence data overlapped with the pandemic period) in our calibration process. The model was calibrated using history matching with emulation and an approximate Bayesian computation Markov chain Monte Carlo method (appendix 3 p 11).^21^ We validated our calibration by comparing estimates of population attributable fraction due to undernutrition from our model to recent estimates.^1^, ^22^

We calibrated 1000 parameter sets, which we used with the mechanistic tuberculosis model to simulate the future and to quantify the uncertainty in tuberculosis natural history (eg, risks of *M tuberculosis* infection and progression to disease) based on previously published literature (appendix 3 p 5).

### Intervention scenarios

The RATIONS trial saw the provision of food baskets (1200 kcal for patients with tuberculosis and 750 kcal for household contacts), alongside micronutrient supplements, to adults on tuberculosis treatment and their household contacts.^9^, ^10^ We considered seven intervention scenarios; this included four mechanisms of action of such a nutritional support intervention (two affecting people with tuberculosis on treatment and two affecting household contacts), a combined scenario of both mechanisms for people with tuberculosis on treatment, a combined scenario of both mechanisms for household contacts, and a combined scenario including all four mechanisms for both people with tuberculosis on treatment and household contacts. Based on the outcomes recorded in the trial, the four mechanisms were as follows: (1) improvements in treatment outcomes for people with tuberculosis on treatment; (2) improvements in BMI for people with tuberculosis on treatment; (3) reductions in tuberculosis disease incidence for household contacts; and (4) improvements in BMI for household contacts. We assumed that improvements in BMI lasted for 2 years. We assumed instant scale-up of the intervention to reach 50% of the adult population (to align with the trial, which enrolled both child and adult household contacts of adult index patients) notified and started on treatment each year from 2023 to 2035, and did not separate public versus private health-care facilities (which can differ in terms of treatment initiation rates, drug susceptibility testing, and treatment outcomes). We assumed that the coverage and quality of existing interventions in the tuberculosis programme remained constant, including coverage of the existing direct benefits transfer scheme, which was implicitly considered in the epidemiological dynamics (due to an absence of efficacy data to do so explicitly).

For improvements in treatment outcomes for people with tuberculosis, the trial did not include a comparator group. We therefore assumed that those who received nutritional support had a reduced hazard of death of 0·67 relative to those who did not receive nutritional support (where the probability of death on treatment was dependent on factors such as age and BMI). This assumption reflects a combination of the 54% of patients in the trial who had weight gain of at least 5% at 2 months, which was associated with a reduced hazard of death (adjusted hazard ratio [HR] 0·39, 95% uncertainty interval [UI] 0·18–0·86), and the remaining 46% of patients who we assumed had an unchanged hazard of death (ie, 1).^10^

Separately, for improvements in BMI for people with tuberculosis on treatment we assumed an increase in BMI in line with the trial^10^ for a proportion of adults on treatment equivalent to the intervention coverage.

For reductions in tuberculosis disease incidence for household contacts, we estimated the number of household contacts (both children and adults) who were expected to develop tuberculosis disease, and reduced this using the trial incident rate ratio (IRR). We estimated the number at risk using the number of households receiving the intervention, the mean household size minus one (the person with tuberculosis on treatment),^14^ and the proportion of those who would be expected to develop tuberculosis disease (3419 [95% UI 1569–5132] per 100 000 population [based on a previous review^23^ using the first 3 years for low-income and middle-income countries only]). We multiplied the number of people at risk by the protective effect of the trial (0·39 [ie, 1 – 0·61]) to identify those who would have gone on to develop tuberculosis disease in the absence of the intervention, and assumed that they remained infected with *M tuberculosis* only.

For improvements in BMI for household contacts we assumed an increase in BMI in household contacts (both adults and children) in line with the trial.^9^ However, household contacts are at an increased risk of tuberculosis disease.^23^ We therefore assumed that 3·1% (95% UI 2·2–4·4) of contacts would have tuberculosis disease, and used the estimated number of household contacts and the intervention coverage to increase the BMI of those with disease in the household.^23^ We then assumed that the remaining contacts had the same BMI distribution (and distribution of *M tuberculosis* infection status other than disease) as the general population, which we again increased in line with the trial results. We assumed a similar increase in weight-for-height and BMI for children and adolescents. We did not consider the impact of tuberculosis screening for existing tuberculosis disease among household contacts.

### Costs

We assumed tuberculosis diagnostic and treatment costs from the health system perspective per patient and episode in previous work.^11^ In addition, from the societal perspective, we accounted for indirect and non-medical patient costs, including productivity loss and transportation while receiving treatment. We assumed a total intervention cost (cost of food basket and delivery) per person receiving tuberculosis treatment of US$92·02, and $33·23 per household contact, based on the cost per month reported in the trial^9^ and assuming 6 months of treatment. Further details are included in appendix 3 (p 16).

### Outcomes

We estimated tuberculosis incidence, mortality rates, and the number of people developing tuberculosis disease and dying due to tuberculosis for each year from 2023 to 2035 for each of the scenarios. For each scenario, we calculated the total tuberculosis costs from the health system and societal perspectives, including indirect and non-medical costs, and the total savings compared with the no-intervention scenario. We discounted both costs and outcomes to 2023 at 3% per year. We calculated the difference in total disability-adjusted life-years (DALYs) from each scenario compared with a scenario with no intervention. For individuals currently with tuberculosis, we used the disability weight (used to weight the magnitude of health loss due to a specific health state, such as tuberculosis disease) from the Global Burden of Diseases, Injuries, and Risk Factors Study 2019 of 0·333 (95% UI 0·224–0·454),^24^ and age-specific life expectancy estimates from the UNDP^25^ to estimate years lived with a disability. We did not incorporate any disability weights for different BMI categories.

We did cost-effectiveness analyses for scenarios delivering the intervention to different groups (those receiving tuberculosis treatment, household contacts of those receiving tuberculosis treatment, or both) and compared incremental cost-effectiveness ratios (ICERs) against three willingness-to-pay thresholds (cost per DALY averted): 1 × gross domestic product (GDP) per capita ($2411 in 2022),^26^ and two India-specific cost thresholds^27^ (upper [$555] and lower [$410] bounds, representing 23% and 17% of GDP per capita, respectively).

### Sensitivity analyses

We varied the duration of protection in our model (ie, how long individuals had an improved BMI or treatment outcome for), comparing whether they returned to their previous state immediately after treatment; remained in an improved state for a mean of 1 year, waning exponentially; remained in an improved state for a mean of 5 years; or remained in an improved state for life.

Separately, we varied the coverage of the intervention, assuming the intervention reached either 20% or 80% of the adult population on treatment each year from 2023 to 2035.

Finally, we varied the intervention impact by BMI status, comparing whether the adjusted HR was higher in those with a low BMI; whether the protective effect of the trial in terms of an IRR for household contacts varied by BMI status as in the trial (where those with a normal BMI had a larger reduced IRR than those who were underweight); or whether both the adjusted HR and the IRR varied by BMI status.

### Role of the funding source

There was no funding source for this study.

## Results

**Figure 1:**
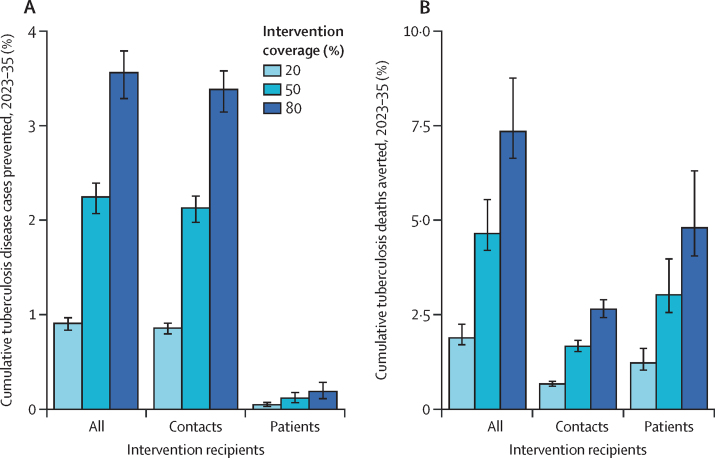
Cumulative proportions of tuberculosis disease cases prevented (A) and deaths averted (B) due to a nutritional support intervention in India between 2023 and 2035, assuming 2 years duration of protection, and considering different recipients and coverage of the intervention Intervention recipients considered were all individuals in tuberculosis-affected households (all), only household contacts of people with tuberculosis (contacts), or only patients with tuberculosis (patients). Intervention coverage specifies the proportion of coverage among adults with tuberculosis receiving treatment.

If nutritional support is provided to 50% of adults notified and started on tuberculosis treatment in India and their household contacts between 2023 and 2035 (ie, the full intervention scenario), we project that a cumulative 880 700 (95% UI 802 700–974 900) fewer people would develop tuberculosis disease and 361 200 (318 000–437 700) fewer deaths would occur as a result (table 1). These values are equivalent to averting 2·2% (2·1–2·4) of all cases of tuberculosis disease and 4·6% (4·2–5·5) of all tuberculosis deaths (figure 1). This effect scales linearly with coverage: at 80% coverage, as many as 1 396 800 (1 273 700–1 545 400) fewer people would develop tuberculosis and 570 900 (502 300–691 400) fewer would die (full results by coverage level are provided in an online repository). Providing nutritional support only to adult patients on tuberculosis treatment with 50% coverage could prevent 46 700 (27 800–71 600) people developing disease, and 234 300 (193 700–305 700) people dying as a result (table 1).

**Table 1:** Health outcomes for nutritional support interventions with 50% coverage of adults receiving tuberculosis treatment in India between 2023 and 2035, assuming 2-year duration of protection

	**No intervention**	**Intervention**						
		Given only to adult patients with tuberculosis			Given only to household contacts			Full intervention§
		Improved outcomes*	Improved BMI†	Combined*†	Reduced incidence‡	Improved BMI†	Combined†‡	
**Total outcomes**								
Incident tuberculosis in 2035, per 100 000 population	157·6 (141·2 to 174·6)	158·0 (141·5 to 175·1)	156·8 (140·5 to 173·5)	157·2 (140·8 to 173·9)	152·6 (136·4 to 169·1)	157·2 (140·8 to 174·1)	152·1 (136·1 to 168·6)	151·6 (135·7 to 168·0)
Tuberculosis deaths in 2035, per 100 000 population	30·5 (27·9 to 33·8)	29·6 (27·1 to 32·8)	30·1 (27·6 to 33·4)	29·3 (26·8 to 32·4)	29·6 (27·0 to 32·8)	30·4 (27·9 to 33·7)	29·5 (27·0 to 32·7)	28·3 (25·9 to 31·4)
DALYs incurred, millions	2212 (2207 to 2218)	2208 (2203 to 2215)	2211 (2206 to 2217)	2207 (2202 to 2214)	2209 (2204 to 2215)	2212 (2207 to 2218)	2209 (2204 to 2215)	2204 (2199 to 2210)
**Outcomes averted**								
Additional cumulative incident tuberculosis, thousands	..	−42·1 (−65·2 to −32·0)	84·2 (66·7 to 121·1)	46·7 (27·8 to 71·6)	777·1 (718·0 to 850·7)	59·2 (51·0 to 69·3)	833·7 (768·7 to 917·3)	880·7 (802·7 to 974·9)
Additional cumulative incident tuberculosis, %	..	−0·1% (−0·2 to −0·1)	0·2% (0·2 to 0·3)	0·1% (0·1 to 0·2)	2·0% (1·8 to 2·1)	0·2% (0·1 to 0·2)	2·1% (2·0 to 2·3)	2·2% (2·1 to 2·4)
Additional cumulative tuberculosis deaths, thousands	..	184·3 (148·8 to 242·4)	59·4 (47·1 to 80·8)	234·3 (193·7 to 305·7)	120·5 (108·9 to 133·8)	8·9 (7·9 to 10·2)	129·2 (116·9 to 143·1)	361·2 (318·0 to 437·7)
Additional cumulative tuberculosis deaths, %	..	2·4% (1·9 to 3·1)	0·8% (0·6 to 1·0)	3·0% (2·6 to 4·0)	1·5% (1·4 to 1·7)	0·1% (0·1 to 0·1)	1·7% (1·5 to 1·8)	4·6% (4·2 to 5·5)
DALYs averted, millions	..	3·7 (2·9 to 4·8)	1·3 (1·0 to 1·6)	4·8 (3·9 to 6·0)	3·1 (2·8 to 3·5)	0·2 (0·1 to 0·2)	3·4 (3·0 to 3·7)	8·0 (7·1 to 9·4)

Values in parentheses are 95% uncertainty intervals. DALYs=disability-adjusted life-years.

* Patients with tuberculosis who received the intervention have improved treatment outcomes (a reduced hazard of death compared with patients with tuberculosis who do not receive the intervention).

† Those receiving the intervention have an increased BMI compared with those who do not receive the intervention.

‡ Household contacts receiving the intervention have a reduced incidence rate ratio for developing tuberculosis disease (ie, they are less likely to progress to tuberculosis disease than household contacts who do not receive the intervention).

§ Given to all individuals in tuberculosis-affected households, assuming the intervention effect has a combined effect of improved outcomes, reduced incidence, and improved BMI.

The duration of protection did not have a significant effect on our results; assuming that improvements in BMI (for both patients and their household contacts) and treatment outcomes would last only while the patient was on treatment reduced the overall effect of the intervention, while waning protection between 5 years and lifelong provided slightly improved outcomes (appendix 3 p 21). Varying the intervention impact by BMI status did not qualitatively change our results; varying the intervention effect on treatment outcomes by BMI reduces the number of tuberculosis deaths averted but makes very little difference to tuberculosis disease prevented, while varying the intervention effect on tuberculosis incidence in household contacts by BMI increases both the number of tuberculosis deaths averted and the number of tuberculosis disease episodes prevented (appendix 3 p 22).

**Figure 2:**
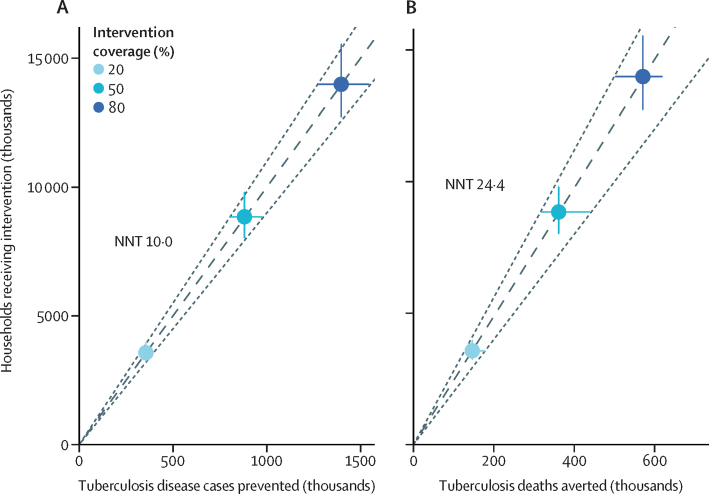
Number of households receiving the nutritional support intervention needed to prevent one person developing tuberculosis disease (A) and one tuberculosis death (B) in India between 2023 and 2035, with varying intervention coverage in adults on treatment NNTs are shown for 50% coverage; for other coverage levels, NNTs were the same to this level of rounding. NNT=number needed to treat.

Assuming 50% coverage of the full intervention, the median number of households that needed to receive the intervention to prevent one person developing tuberculosis disease was 10·0, and to prevent one person dying as a result was 24·4 (table 2). The number needed to treat remained stable irrespective of intervention coverage, with some variability due to model uncertainty (figure 2). Reducing the duration of protection increased the number needed to treat.

**Table 2:** Resources required for nutritional support interventions with 50% coverage of adults receiving tuberculosis treatment in India between 2023 and 2035, assuming 2-year duration of protection

	**No intervention**	**Intervention**		
		Given only to patients with tuberculosis	Given only to household contacts	Full intervention*
**Total resources, US$ millions**				
Average total health system cost	8618 (7990–9355)	9281 (8598–10 093)	9313 (8624–10 127)	9967 (9220–10 846)
Average total societal cost	11 967 (11 096–12 991)	12 623 (11 695–13 719)	12 610 (11 677–13 702)	13 256 (12 271–14 417)
**Additional resources required**				
Additional health system cost†, US$ millions	..	664 (605–733)	696 (625–771)	1349 (1221–1492)
Additional societal cost†, US$ millions	..	656 (599–724)	643 (574–715)	1289 (1163–1426)
People with tuberculosis receiving support, thousands	..	8947 (8141–9937)	..	8829 (8033–9807)
Household contacts receiving support, thousands	..	..	30 101 (27 384–33 450)	30 018 (27 312–33 344)
**Number of households needed to treat**				
To avert one case of tuberculosis	..	191·6	10·6	10·0
To avert one death due to tuberculosis	..	38·2	68·5	24·4
**Cost-effectiveness, US$ per DALY averted**				
Incremental health system cost-effectiveness†	..	139 (113–167)	..	167 (147–187)
Incremental societal cost-effectiveness†	..	137 (112–164)	..	192 (166–218)

Values in parentheses are 95% uncertainty intervals. All costs are discounted at 3% per year.

* Given to all individuals in tuberculosis-affected households.

† Compared with no intervention.

**Figure 3:**
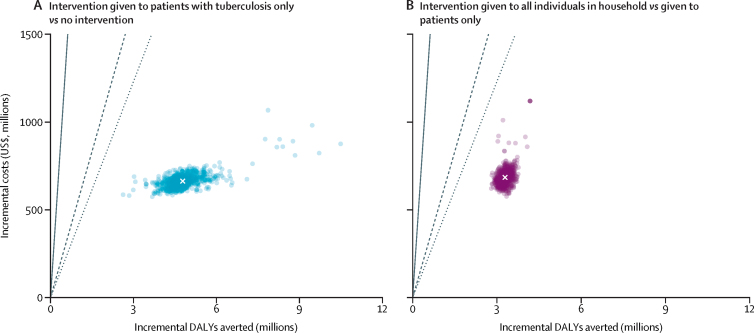
Incremental cost-effectiveness of the nutritional support intervention for patients with tuberculosis (A) and for patients and their household contacts (B) from a health system perspective at 50% coverage Three willingness-to-pay thresholds are shown, based on 1 × Indian GDP per capita in 2022 ($2411);^26^ 23% of Indian GDP per capita ($555); and 17% of Indian GDP per capita ($410).^27^ DALYs=disability-adjusted life-years. GDP=gross domestic product.

From a health system perspective, providing the nutritional support intervention only for patients with tuberculosis is likely to be cost-effective at most willingness-to-pay thresholds, with an ICER of $139 (95% UI 113–167) per DALY averted (table 2; figure 3) and total budget impact (additional health system cost) of $664 million (605–733; appendix 3 p 24). Compared with providing the intervention only to patients with tuberculosis, providing it additionally to household contacts of patients with tuberculosis is also likely to be cost-effective at most thresholds, with an ICER of $208 (181–234) per DALY averted and a budget impact of an additional $685 million (615–759 million). Both the intervention and treatment costs reduce over time. Compared with no intervention, provision of the full intervention has an ICER of $167 (147–187) per DALY averted, which remains consistent with increasing coverage. Results are qualitatively similar from a societal perspective (see online repository for full results).

## Discussion

Compared with a baseline with no nutritional intervention for patients with tuberculosis or their contacts, at 50% coverage of those on tuberculosis treatment (around 23% of incident tuberculosis-affected households), providing nutritional support to people with tuberculosis would prevent 234 300 deaths due to tuberculosis in India between 2023 and 2035, and prevent 46 700 people from developing the disease. Such an intervention would cost $664 million, with an ICER of $139 per DALY averted. Extending support to household contacts would prevent a total of 361 200 tuberculosis deaths and 880 700 cases of tuberculosis diseases, equivalent to averting 2·2% of all tuberculosis deaths and 4·6% cases of tuberculosis disease from 2023 to 2035. The additional cost would be $685 million, with an ICER of $208 per DALY averted. To prevent one person developing tuberculosis or one person dying from tuberculosis would require nutritional support under the full intervention scenario to 10·0 and 24·4 tuberculosis-affected households, respectively.

The effect of the intervention is constrained by two elements. First, the proportion of *M tuberculosis* transmissions that occur outside of the household, particularly in a setting with a high tuberculosis burden such as India, limits the amount of tuberculosis disease as well as subsequent tuberculosis deaths that can be prevented through reduced progression. Second, the proportion of tuberculosis deaths that occur in those who remain undiagnosed with tuberculosis disease limits the tuberculosis deaths that can be averted through improved nutrition while on treatment. As a result, an intervention targeting only those on tuberculosis treatment and their households is able to avert a relatively low proportion of tuberculosis incidence and mortality, as compared with a population-level intervention to improve nutrition, which could have a substantially larger effect size (albeit with a much larger associated cost). By contrast, a nutritional intervention targeted towards those at highest risk, such as household contacts or those with undernutrition, particularly in a resource-constrained setting such as India, offers the opportunity to maximise cost-effectiveness within a feasible budget, improving population-level equity and allowing for a meaningful reduction in tuberculosis burden among those most susceptible to the disease. A targeted intervention in particular offers fewer resource allocation challenges, although the potential opportunity costs are important to consider within the context of wider programmatic activities.

The number needed to treat in our results was consistently substantially smaller than in the trial (30 households to prevent one incident case),^9^ a result of future transmission averted in our model. The targeted nature of the intervention, focused on tuberculosis-affected households at a higher risk of both tuberculosis disease and poor outcomes, meant that the intervention was likely to see a much higher return for effort than a more general population-based intervention, and supports evidence of its cost-effectiveness. Our results showed that an intervention with 50% coverage of adult patients with tuberculosis and their households would avert around 2% of cumulative incidence between 2023 and 2035. This is similar in scale to estimates for a household-level intervention in a previous study,^28^ which suggested an intervention improving BMI for all undernourished household contacts would see a 4·5% reduction in cumulative tuberculosis incidence and a 4·8% reduction in mortality between 2023 and 2035 given 100% coverage. Our results also showed a similar cost-effectiveness to that of recent studies, although somewhat improved due to additional transmission averted.^29^

Our model was limited by a number of assumptions for the intervention effect. A lack of comparator group for index patients in the trial means that we might have overestimated improvements in outcomes due to weight gain, which could also have occurred in part due to tuberculosis treatment.^3^ Including both improvements in BMI and improvements in treatment outcomes might also lead to double-counting of the impact of the intervention effect, although the effect size of this is likely to be small. A lack of private treatment pathways in our model might have overestimated the achievable coverage of the intervention, and, similarly, our assumption of instant scale-up is highly optimistic, while other improvements in the programme over time would also reduce the potential intervention effect. We also did not explicitly consider drug-resistant tuberculosis in our analysis; notably, there is an urgent need for additional data in this area due to a higher prevalence of both undernutrition and poor treatment outcomes for drug-resistant tuberculosis.^1^ On one hand, costs associated with the provision of nutritional support for a longer period (corresponding to the extended treatment period for drug-resistant tuberculosis, often around 9–24 months) will reduce the cost-effectiveness of the intervention. On the other hand, these costs will likely be more than offset by the additional cost-savings accrued with every drug-resistant tuberculosis treatment regimen averted.

By contrast, extending the intervention beyond adults on tuberculosis treatment to children and adolescents would increase the potential impact. Variation in both prices and implementation across the country could also lead to important regional differences not considered here, as well as indirect benefits to the community of sourcing food locally. It is also potentially problematic to generalise from the trial setting (a single Indian state with a high burden of both undernutrition and tuberculosis) to the country as a whole. However, when we varied the intervention effect by BMI status (which would to some extent account for regional differences in the burden of undernutrition), we saw a larger impact of the intervention, suggesting that our results are still likely to be valid. It would also be inadvisable to extrapolate our findings to individual states or regions only due to the wide variance in the burden of undernutrition and tuberculosis, as well as in access to health care and the relative importance of different settings of transmission. We also did not consider any potential case-finding component of the intervention, in which household contacts with prevalent tuberculosis might have been more likely to have been diagnosed due to the intervention (assuming good diagnostic performance), which would have increased the intervention effectiveness. Most importantly, however, we did not consider the wider, non-tuberculosis-specific benefits of the intervention and improvements in weight in tuberculosis-affected households, such as improved general health and ability to function, and reductions in post-tuberculosis lung damage. These benefits could potentially further increase the intervention impact and cost-effectiveness substantially.

The RATIONS study was the first adequately powered, randomised trial to assess the combined effects of macronutrient and micronutrient interventions on tuberculosis incidence, making it uniquely suited for parametrising our model. However, it is essential that further research in diverse contexts be conducted to confirm and validate these promising findings. To ensure maximal uptake, food baskets should be designed through community-engaged research, incorporating the dietary practices, preferences, and cultural norms of the target population. Additionally, community-specific nutritional counselling can serve as an enabler to ensure the optimal use of the food baskets, although further research is needed to evaluate the additive effect and cost-effectiveness of providing nutritional counselling alongside food support interventions.

Our results show that widespread coverage of a nutritional support intervention for patients with tuberculosis and their household contacts in India could prevent nearly 900 000 people developing tuberculosis disease (>2% of cases) and over 350 000 tuberculosis deaths (nearly 5%) by 2035. Such an intervention could have a substantial effect on the tuberculosis epidemic in India, reducing both incident tuberculosis disease and tuberculosis deaths, and is likely to be cost-effective at most willingness-to-pay thresholds. These results are likely to be a considerable underestimate, as considering the wider health benefits beyond tuberculosis would only further increase the benefit and cost-effectiveness of the intervention. There is an urgent need to generate further evidence, including implementation research to design intervention approaches and evaluate their implications, and from a wider variety of settings, to support rapid translation of this intervention into policy and practice.

### Contributors

### Equitable partnership declaration

### Data sharing

The analytic code and additional model results for this study are available online at https://doi.org/10.5281/zenodo.14160778.

## Declaration of interests

AB is an unpaid member of the Scientific Advisory Committee, ICMR–National Institute of Nutrition, Hyderabad; and of the Strategic Technical Advisory Group on Tuberculosis, WHO South-East Asia Region and WHO, Geneva. All other authors declare no competing interests.

## Supplementary Materials

Bangla translation of the abstract

Hindi translation of the abstract

Supplementary appendix 3

Equitable Partnership Declaration
