# Supplementary material for: Estimating the epidemiological and economic impact of providing nutritional care for tuberculosis-affected households across India: a modelling study
Source: Lancet Glob Health. 2025 Jan 14;13(3):e488–96. doi: 10.1016/S2214-109X(24)00505-9 (PMC11865009; doi:10.1016/S2214-109X(24)00505-9)
Supplement: Bangla translation of the abstract [file mmc1.pdf]

THE LANCET
Global Health

Supplementary appendix 1

This translation in Bangla was submitted by the authors and we reproduce it as supplied. It has not been peer reviewed. *The Lancet*'s editorial processes have only been applied to the original in English, which should serve as reference for this manuscript.

'এই [বাংলায়] অনুবাদটি লেখকরা জমা দিয়েছিলেন এবং এটি যেমনভাবে দেওয়া হয়েছে আমরা সেইভাবেই পুনরায় বর্ণনা করছি। এটি কোনো সমকক্ষ ব্যক্তি পর্যালোচনা করেননি। দ্য ল্যানসেট-এর সম্পাদকীয় প্রক্রিয়াগুলি শুধুমাত্র মূল ইংরেজিতে প্রয়োগ করা হয়েছে, যা এই পান্ডুলিপির রেফারেন্স হিসাবে কাজ করবে।'

Supplement to: McQuaid CF, Clark RA, White RG, et al. Estimating the epidemiological and economic impact of providing nutritional care for tuberculosis-affected households across India: a modelling study. *Lancet Glob Health* 2025; published online Jan 14. https://doi.org/10.1016/S2214-109X(24)00505-9.

পটভূমি

বিশ্বব্যাপী যক্ষ্মার প্রায় ২০%-এর সঙ্গে অপুষ্টির সম্পর্ক রয়েছে, ভারতে তা এক-তৃতীয়াংশের বেশি। যক্ষ্মা-প্রভাবিত পরিবারগুলোর জন্য পুষ্টি বিষয়ক হস্তক্ষেপের নীতিগতভাবে অগ্রাধিকার পাওয়া উচিত, কিন্তু এর মহামারীবিদ্যাগত ও অর্থনৈতিক প্রভাব সম্পর্কে আমাদের জ্ঞান সীমিত। আমরা এমন একটি হস্তক্ষেপের জনসংখ্যা-স্তরের মহামারীবিদ্যাগত ও অর্থনৈতিক প্রভাব নির্ধারণ করতে চেয়েছি।

পদ্ধতি

আমরা পূর্বে প্রকাশিত একটি বয়সভিত্তিক, বিভাগীয় সংক্রমণ মডেল ব্যবহার করেছি, যা ভারতে যক্ষ্মার জন্য প্রযোজ্য, এবং এতে বিএমআই (বডি মাস ইনডেক্স) সম্পর্কিত স্তর যুক্ত করেছি, যা রোগের অগ্রগতি ও চিকিৎসার ফলাফলের সঙ্গে সম্পর্কিত। আমরা সাম্প্রতিক কালে পরিচালিত একটি পরীক্ষা থেকে প্রাপ্ত ফলাফল ব্যবহার করেছি, যেখানে যক্ষ্মা চিকিৎসা শুরু করা ব্যক্তিদের এবং তাদের পরিবারের সদস্যদের খাদ্য সহায়তা প্রদান করা হয়েছিল (রোগীর জন্য ১২০০ ক্যালরি এবং পরিবারের সদস্যদের জন্য ৭৫০ ক্যালরি)। এই তথ্য ব্যবহার করে আমরা হস্তক্ষেপের প্রভাব এবং খরচের হিসাব করেছি এবং স্বাস্থ্য ব্যবস্থার দৃষ্টিকোণ থেকে খরচ-কার্যকারিতা মূল্যায়ন করেছি। আমরা অনুমান করেছি যে, ২০২৩ থেকে ২০৩৫ সাল পর্যন্ত এই হস্তক্ষেপে যক্ষ্মা রোগের ঘটনা ও মৃত্যু কতটা প্রতিরোধ করা সম্ভব।

ফলাফল

যক্ষ্মার চিকিৎসা নেওয়া প্রাপ্তবয়স্কদের এবং তাদের পরিবারের ৫০% কভারেজ (ভারতে যক্ষ্মা-প্রভাবিত পরিবারগুলোর প্রায় ২৩%) ধরে নেওয়া হলে, পুষ্টি সহায়তা প্রদান হস্তক্ষেপটি ২০২৩ থেকে ২০৩৫ সালের মধ্যে ৩,৬১,২০০ (৯৫% অনিশ্চয়তা সীমা: ৩,১৮,০০০–৪,৩৭,৭০০) যক্ষ্মা মৃত্যুর ঘটনা এবং ৮,৮০,৭০০ (৮,০২,৭০০–৯,৭৪,৯০০) রোগের ঘটনা প্রতিরোধ করতে পারে। এটি প্রায় ৪.৬% (৪.২–৫.৫) যক্ষ্মা মৃত্যুর এবং ২.২% (২.১–২.৪) রোগের ঘটনা প্রতিরোধের সমান। এতে স্বাস্থ্য ব্যবস্থার অতিরিক্ত খরচ হবে ১৩৪৯ মিলিয়ন মার্কিন ডলার (১২২১–১৪৯২), এবং প্রতিবন্ধকতার জন্য সামঞ্জস্য করা জীবন-বর্ষ (DALY) প্রতিরোধে আনুমানিক খরচ-কার্যকারিতা অনুপাত হবে ১৬৭ মার্কিন ডলার (১৪৭–১৮৭)। একটি যক্ষ্মা মৃত্যু প্রতিরোধ করতে গড়ে ২৪.৪ পরিবার এবং একটি যক্ষ্মা রোগের ঘটনা প্রতিরোধ করতে গড়ে ১০টি পরিবারকে চিকিৎসা দিতে হবে।

উপসংহার

যক্ষ্মা-প্রভাবিত পরিবারের জন্য একটি পুষ্টি হস্তক্ষেপ ভারতে উল্লেখযোগ্য পরিমাণে যক্ষ্মা রোগ এবং মৃত্যু প্রতিরোধ করতে পারে এবং শুধুমাত্র যক্ষ্মা-সম্পর্কিত সুবিধার ভিত্তিতেই এটি অত্যন্ত কার্যকরী হবে।
