# Supplementary material for: Estimating the epidemiological and economic impact of providing nutritional care for tuberculosis-affected households across India: a modelling study
Source: Lancet Glob Health. 2025 Jan 14;13(3):e488–96. doi: 10.1016/S2214-109X(24)00505-9 (PMC11865009; doi:10.1016/S2214-109X(24)00505-9)
Supplement: Hindi translation of the abstract [file mmc2.pdf]

THE LANCET
Global Health

Supplementary appendix 2

This translation in Hindi was submitted by the authors and we reproduce it as supplied. It has not been peer reviewed. *The Lancet*'s editorial processes have only been applied to the original in English, which should serve as reference for this manuscript.

हिंदी में यह अनुवाद लेखकों द्वारा प्रस्तुत किया गया था और हम इसे जैसे उपलब्ध कराया गया वैसे पुन: पेश करते हैं। इस पर सहकर्मी की समीक्षा नहीं की गई है। लैंसेट की संपादकीय प्रक्रियाओं को केवल अंग्रेजी में मूल पर लागू किया गया है, जो इस पांडुलिपि के संदर्भ के रूप में काम आना चाहिए।

Supplement to: McQuaid CF, Clark RA, White RG, et al. Estimating the epidemiological and economic impact of providing nutritional care for tuberculosis-affected households across India: a modelling study. *Lancet Glob Health* 2025; published online Jan 14. https://doi.org/10.1016/S2214-109X(24)00505-9.

बैकग्राउंड
विश्व में तपेदिक (टीबी) की लगभग 20% घटनाएँ कुपोषण के कारण होती हैं, और भारत में यह लगभग एक तिहाई से अधिक है। टीबी प्रभावित परिवारों के लिए पोषण संबंधी हस्तक्षेप को नीतिगत प्राथमिकता देना आवश्यक है, परंतु हमारी विज्ञान और आर्थिक प्रभाव की समझ अभी सीमित है।

मेथड्स
हमने बॉडी मास इंडेक्स के स्तर से जुड़े टीबी का एक ट्रांसमिशन मॉडल विकसित किया है और उकसों रोग की प्रगति और उपचार के परिणामों से जोड़ा है। हमने हाल के एक परीक्षण (रैशन ट्रायल) के परिणामों का इसमें उपयोग किया है। इनसे टीबी का इलाज शुरू करने वाले लोगों और उनके परिवारों को पोषण संबंधी सहायता के प्रभाव और लागत के अनुमानों का आंकलन यहाँ प्रस्तुत किया है।

निष्कर्ष
बिना किसी पोषण संबंधी हस्तक्षेप वाली बेसलाइन की तुलना में, 50% वयस्क टीबी मरीज (~23% नए टीबी) और उनके परिवारों को पोषण सहायता से 361,200 (318,000-437,700) टीबी से होने वाली मौतों और 880,700 (802,700-974,900) बीमारी को 2023-2035 दौरान रोका जा सकता है। यह लगभग 20 में से एक (4.2%-5.5%) टीबी से मृत्यु को टालने के बराबर है और 50 में से एक (2.1%-2.4%) टीबी रोकने बराबर है। स्वास्थ्य सिस्टम की अतिरिक्त लागत $1,349 मिलियन (1,221-1,492 मिलियन) होगी। प्रति विकलांगता-समायोजित जीवन वर्ष (डिसेबिलिटी अडजसटेड लाइफ इयर्स) टालने की वृद्धिशील लागत (इन्क्रमेनल कोस्ट ईफेक्टिव्नेस रेशो) $167 (147-187) होगी। एक टीबी से मृत्यु को रोकने के लिए औसतन 24.4 और एक टीबी

प्रकरण रोकने के लिए 10.0 परिवारों को पोषण प्राप्त करने की आवश्यकता होगी। यदि 80% तक कवरेज (37% टीबी) कर पाए, तो 14 में से एक मृत्यु और 30 टीबी प्रकरणों को रोका जा सकता है। इससे $2,139 मिलियन की कुल वृद्धिशील लागत (इन्क्रमेनल कोस्ट) हो सकती है।

<u>कन्क्लूसन</u>

टीबी से प्रभावित परिवारों के लिए पोषण संबंधी हस्तक्षेप से काफी मात्रा में टीबी को रोका जा सकता है और यह प्रभावशाली और किफायती भी साबित हो सकता है।
