# Supplementary material for: Estimating the epidemiological and economic impact of providing nutritional care for tuberculosis-affected households across India: a modelling study
Source: Lancet Glob Health. 2025 Jan 14;13(3):e488–96. doi: 10.1016/S2214-109X(24)00505-9 (PMC11865009; doi:10.1016/S2214-109X(24)00505-9)
Supplement: Supplementary appendix 3 [file mmc3.pdf]

THE LANCET
Global Health

Supplementary appendix 3

This appendix formed part of the original submission and has been peer reviewed. We post it as supplied by the authors.

Supplement to: McQuaid CF, Clark RA, White RG, et al. Estimating the epidemiological and economic impact of providing nutritional care for tuberculosis-affected households across India: a modelling study. *Lancet Glob Health* 2025; published online Jan 14. https://doi.org/10.1016/S2214-109X(24)00505-9.

Supplementary material for *Estimating the epidemiological and economic impact of providing nutritional care for TB-affected households across India*

C. Finn McQuaid, Rebecca A. Clark, Richard G. White, Roel Bakker, Peter Alexander, Roslyn Henry, Pranay Sinha, Madhavi Bhargava, Anurag Bhargava, Rein M.G.J. Houben

SUPPLEMENTARY METHODS 2
1. TB transmission model 2
1.1 TB model structure and natural history 2
1.2 Incorporating BMI into the TB model 8
1.3 TB model simulation, calibration, and validation 11
2. Climate and food systems model 14
2.1 LandSyMM 14
2.2 Population weight distributions 15
3. Economic analysis methods 16
3.1 Calculation of disability-adjusted life years 16
3.2 Tuberculosis-related and intervention costs 16
3.3 Cost-effectiveness analysis and willingness-to-pay thresholds 17
SUPPLEMENTARY RESULTS 18
4. Epidemiological trends in the *No Intervention* baseline scenario 18
5. Intervention scenario results 21
5.1 Health impact results 21
5.2 Cost-effectiveness results 23
5.3 Budget Impact results 24
REFERENCES 25

SUPPLEMENTARY METHODS

1. TB transmission model

We created an age-stratified compartmental differential equation model of tuberculosis (TB) in India, including dimensions for age, TB natural history, and body mass index (BMI). The age structure was identical to that included in Clark et al., 2023.[1] Minor modifications from the Clark et al. natural history structure are described below in section 1.1, and extensions to include four BMI strata, as well as differences in TB progression and treatment, are in section 1.2. A summary of the model process is visualised in Figure S1 below.

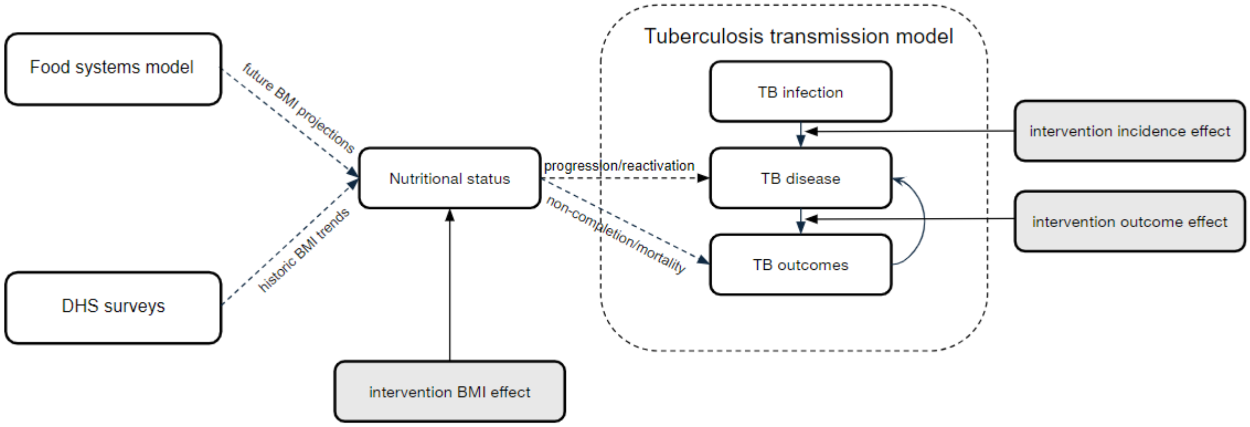

Figure S1 Simplified model process

1.1 TB model structure and natural history

The TB natural history structure with eight compartments is shown in Figure S2 and has been described previously.[1] The latency structure in this model demonstrated a progressive loss of ability to reactivate, with the reactivation rate in the Latent-Fast compartment greater than in Latent-Slow and greater still than in Latent-Zero, where we assumed the rate of reactivation is 0. We did not explicitly consider a self-clearance compartment. We assumed that those in Latent-Fast could fast progress to subclinical disease, or continue to remain latent and transition to Latent-Slow. There was no direct transition from Latent-Fast to Latent-Zero.

Parameters used in the natural history model structure are provided in Table S1 below, along with their definitions, sources, and information on whether the parameter was fixed or varied (as well as whether they were varied by age or time) during calibration. The parameter ranges provided for the TB natural history parameters were priors fitted during calibration in a Bayesian analysis. We assumed that all values within

the prior range were equally likely. The prior ranges were pre-specified based on literature review and were reviewed as new data became available.

We assumed that aspects of TB natural history and mortality varied by age. This was implemented by stratifying certain natural history parameters by age and applying age-specific prior ranges and relative constraints during calibration.[2] We explicitly model single year age groups from age 0 to 79 years, and categories for ages 80–89 and 90–99. Table S2 describes the method used to operationalise the age-varying differences in TB natural history parameters between adults, defined as all ages greater than and equal to 15 years, and children, defined as all ages less than 15 years. Due to limited data, we assume homogeneous natural history parameters for all single year age groups within these broader categorizations of adults and children. For the rate per year of reactivation, relapse, and fast progression to tuberculosis disease, we assumed that the rate for children was less than that for adults. For mortality rates, we assumed the opposite: the rate for children was higher than that for adults. Steps for calculating TB treatment initiation, treatment completion, non-completion, and mortality rates, as well as accounting for public and private sector treatment are described in the Supplementary Material for Clark et. al.[1]

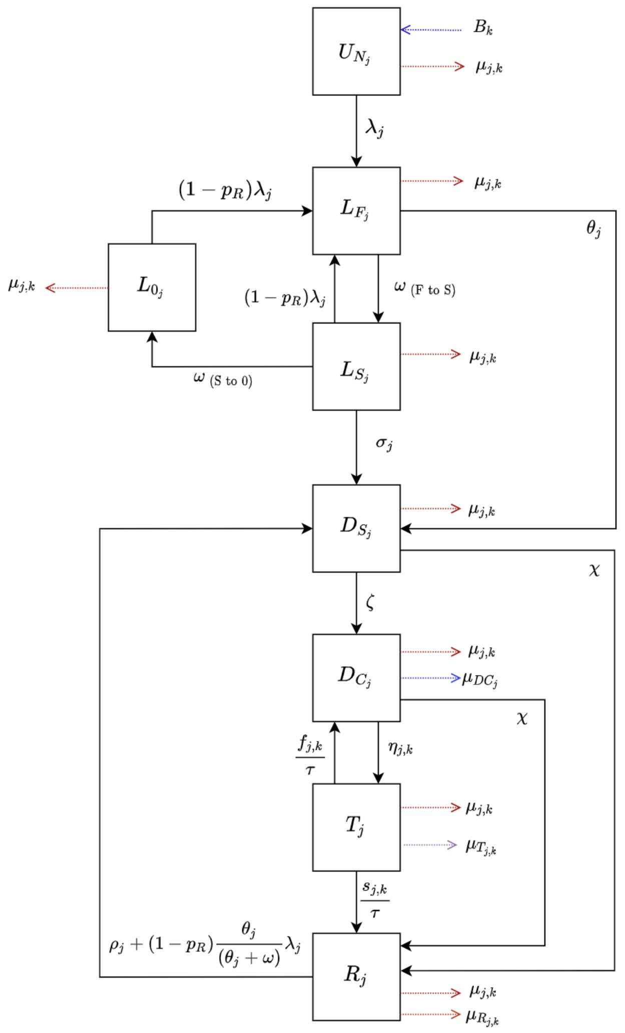

Figure S2 Tuberculosis natural history model structure

Subscript j represents parameters that vary by age, and subscript k represents parameters that vary over time.

Abbreviations: U_N = Uninfected-Naive; L_F = Latent-Fast; L_S = Latent-Slow; L_0 = Latent-Zero, D_S = Subclinical Disease; D_C = Clinical Disease; T = On-Treatment; R = Recovered.

Table S1 India national model parameter values and sources

Description	Units	Symbol	Prior	Fixed or Varying During Calibration	Age Varying	Time Varying	Source
Births and deaths (excluding on-treatment mortality)							
Birth rate	Per year	B_k	UN World Population Prospects estimates and projections	Fixed	No	Yes	[3]
Background mortality rate	Per year	$\mu_{j,k}$	Calculated in the model from United Nations population estimates and projections	Fixed	Yes, age specific mortality rates from demographic dataset	Yes	[3]
Mortality rate for clinical tuberculosis disease	Per person per year	μ_{DC_j}	(0–0.178)	Varying	Yes, value for children is greater than value for adults	No	[4]
Mortality rate post-tuberculosis disease	Per person per year	μ_{R_j}	$0.22\mu_{j,k}$	Fixed relationship	Yes because $\mu_{j,k}$ varies	Yes because $\mu_{j,k}$ varies	[5]
Natural History							
Force of infection	Per year	λ_j	Fitted	Fixed Equation	Yes, age specific contact rates [6]	No	*Calculated*
Probability of transmission per infectious contact	-	p_T	(0–0.0068)	Varying	No	No	*Assumed*
Fraction of total tuberculosis that is extrapulmonary	-	ep	0.222	Fixed	No	No	[7,8]
Infectiousness of subclinical relative to clinical tuberculosis	-	r	0.83	Fixed	No	No	[9]

Rate of fast progression to disease, by age	Per person per year	θ_j	(0.0696–0.111)	Varying	Yes; retained if value for children was **less** than value for adults.	No	[10]
Rate from L_F to L_S	Per person per year	ω_{FS}	0.5	Fixed	No	No	*Defined*
Rate of reactivation from L_S, by age	Per person per year	σ	(0.000135–0.00113)	Varying	Yes; retained if value for children was **less** than value for adults.	No	[10]
Rate from L_S to L_0	Per person per year	ω_{S0}	(0.0254–0.0467)	Fixed	No	No	[10]
Rate of progression from D_S to D_C	Per person per year	ζ	(0–12)	Varying	No	No	*Assumed*
Rate of natural cure from D_C and D_S	Per person per year	χ	(0.10–0.25)	Varying	No	No	[11,12]
Rate of relapse from R, by age	Per person per year	ρ_j	(0.0001–0.07)	Varying	Yes; retained if value for children was **less** than value for adults.	No	[13–15]
Protection Parameters							
Protection from reinfection L_S, L_F, L_0, R	-	p_R	(0.60–0.85)	Varying	No	No	[11,12,16–18]
Access-to-care parameter	-	p_E	(0–1)	Varying	No	No	*Assumed*

Table S2 Age-varying natural history and treatment parameters

Parameter	Range	Age Varying Description	Age Scaling Parameter	Adults (θ_{A15})	Children (θ_{A0})
θ_j<br>Rate per year of fast progression	$(0.0696–0.111)$	Retained if value for children was **less** than value for adults	Sample j_1 from $(0–1)$	Sample θ_{A15} from $(0.0696–0.111)$	$\max(0.0696, \theta_{A15} \times j_1)$
σ_j<br>Rate per year of reactivation	$(0.000135–0.00113)$	Retained if value for children was **less** than value for adults	Sample j_2 from $(0–1)$	Sample σ_{A15} from $(0.000135–0.00113)$	$\max(0.000135, \sigma_{A15} \times j_2)$
ρ_j<br>Rate per year of relapse	$(0.01–0.07)$	Retained if value for children was **less** than value for adults	Sample j_3 from $(0–1)$	Sample ρ_{A15} from $(0.01–0.07)$	$\max(0.01, \rho_{A15} \times j_3)$
μ_{DC_j}<br>Clinical TB mortality rate per year	$(0–0.178)$	Retained if value for children was **greater** than value for adults	Sample S_{Age} from $(0–1)$	$\mu_{DC_{A0}} \times S_{Age}$	Sample $\mu_{DC_{A0}}$ from $(0–0.178)$
$\mu_{T_j} = \frac{\kappa_j}{\tau}$<br>On-treatment mortality rate per year	$(0–0.270)$	Retained if value for children was **greater** than value for adults	Sample S_{Age} from $(0–1)$	$\frac{\kappa_{A0}}{\tau} \times S_{Age}$	Sample κ_{A0} from $(0–0.135)$

1.2 Incorporating BMI into the TB model

BMI strata

We defined four BMI strata to include as an additional dimension in the TB transmission model: moderate to severe thinness (BMI < 17.0 kg/m^2), mild thinness (17.0 kg/m^2 ≤ BMI < 18.5 kg/m^2), normal BMI (18.5 kg/m^2 ≤ BMI < 25.0kg/m^2) and overweight to obese BMI (BMI ≥ 25.0 kg/m^2).

Distribution of the population in each BMI strata

The age-standardised proportion of the adult population >19 years within each strata was taken from the Global Health Observatory for the years 1975–2016.[19] This consisted of the proportion of the population overweight (BMI ≥ 25.0 kg/m^2) or thin (BMI < 18.5 kg/m^2), from which the proportion of normal weight was calculated. The proportion thin was separated into moderate to severe thinness and mild thinness based on trends in the ratio of each in the India Demographic and Health Surveys from 2005–2006 and 2015–2016,[20–22] weighted by the male:female ratio from UN population estimates[3] and using the survey package in R.[23] The male:female ratio was used to estimate the proportion of the population in each year with mild thinness compared to moderate to severe thinness (which is reported in the Demography and Health Surveys by sex). We then assume a linear trend in the change in proportion with thinness who had mild thinness between the two surveys.

To estimate the proportion of children and adolescents in each BMI strata, we again used the Global Health Observatory.[19] We separated children aged 0–4 years into BMI strata based on standard deviations (SD) for weight-for-height of severe wasting (weight-for-height < -3SD), wasting (-3SD ≤ weight-for-height < -2SD), normal (-2SD ≤ weight-for-height < +2SD) and overweight (weight-for-height ≥ +2SD), aligning with our four BMI strata. We note that estimates were only available for normal, wasting and severe wasting for the years 1999, 2006, 2014, 2015 and 2017, while for overweight these were available for 1994–2019.

We separated children and adolescents aged 5–19 years also based on SD, but in this instance for BMI, of thinness (BMI < -2SD), normal (-2SD ≤ BMI < +1SD) and overweight (BMI ≥ +2SD). These estimates were available for 1975–2016. The proportion thin was separated as for adults, into moderate to severe thinness (BMI < -3SD) and mild thinness (-3SD ≤ BMI < -2SD) based on trends in the ratio of each in adults in Demographic and Health Surveys.

Varying parameters by BMI strata

We re-estimated the relationship between BMI and TB incidence from Lönnroth et al,[24] using updated data from Cegielski et al,[25] and assuming a uniform distribution for BMI within each BMI strata. We used this to fit a log-linear model with a dependent variable of TB incidence, an independent variable of BMI, and a categorical variable for the studies reported in Lönnroth et al.[24]

Using the log-linear model, the BMI distributions from above, and estimated population-level TB incidence in 2015 (TB incidence in 2019–2021 may have been potentially affected by COVID-19-associated disruptions, while 2005–2006 did not include primary sampling units in IPUMS and 1998–1999 did not include data for men),[20–22] we estimated the expected TB incidence for each BMI strata weighted by the female:male ratio. We used this to calculate the risk ratio for TB in each strata compared to the strata with normal BMI, assuming that differences in risk of incident TB were a direct result of differences in rate of progression or reversion to TB disease. We estimated the mean and uncertainty intervals for this from 10,000 runs of the process (Table S3). If we used BMI categories and reference groups similar to Oxlade et al,[26] we obtain similar risk ratios for progression to that study; 4.96 [4.08–5.83] for the first strata with BMI ≤ 17.8 kg/m^2, 3.43 [2.96–3.90] for the second with 17.8 kg/m^2 ≤ BMI < 19.64 kg/m^2, and 2.38 [2.14–2.62] for the third with 19.64 kg/m^2 ≤ BMI < 22.25 kg/m^2, all compared to the reference strata with BMI ≥ 22.25 kg/m^2.

Treatment outcome estimates were calculated using data collected from adults with microbiologically confirmed TB in five sites of the Regional Prospective Observational Research on Tuberculosis (RePORT) India consortium from 2015–2019.[27] The primary exposure was BMI at treatment initiation and the outcome was a composite of death, treatment failure, and relapse/recurrence. We conducted multivariable analysis using Poisson regression, in which we used person-time as an offset to calculate adjusted incidence rate ratios (aIRR). We calculated person-time from the time of treatment initiation to the occurrence of the first mutually exclusive outcome of interest (failure/recurrence/death), loss to follow-up, or until right-censoring at 24 months of follow-up. In multivariable analysis, we included age, sex, and cough duration in the multivariable a priori and included other potential confounders based on prior literature that were significant at the p<0.2 level. The original publication[27] calculated risk for different BMI categories than the ones required for this model. We recalculated the aIRR for the following BMI categories: moderately to severely thin (BMI < 17.0 kg/m^2), mildly thin (17.0 kg/m^2 ≤ BMI < 18.5 kg/m^2), normal (18.5 kg/m^2 ≤ BMI < 25.0kg/m^2) and overweight to obese (BMI ≥ 25.0 kg/m^2). Due to the small sample size, our results show limited to no evidence for a difference in outcomes except for the moderately to severely thin category.

We assumed that the risk ratios for disease progression, reversion, and treatment outcomes were different by BMI strata, and were applied identically to adults, adolescents, and children, due to a lack of data for the latter two age groups.

Table S3 Risk ratios for natural history and treatment outcome parameters by BMI strata. Parameters affected correspond to those in Table S2.

**BMI strata**	**BMI interval**	**Risk ratio [95% CI] applied to all progression & reactivation parameters θ_j, σ_j, ρ_j**	**Risk ratio [95% CI] for treatment non-completion parameter f_j**	**Risk ratio [95% CI] for on-treatment mortality parameter μ_{Tj}**
Moderate to severely thin	<17.0 kg/m^2	1.73 [1.57–1.89]	1.58 [1.09–2.31]	3.38 [1.84–6.48]
Mildly thin	17.0–18.4 kg/m^2	1.43 [1.32–1.54]	1.14 [0.72–1.79]	0.75 [0.24–1.93]
Normal	18.5–24.9 kg/m^2	REF	REF	REF
Overweight to obese	>25.0 kg/m^2	0.26 [0.21–0.31]	0.82 [0.34–1.69]	1.14 [0.26–3.47]

1.3 TB model simulation, calibration, and validation

We followed the same process for model simulation and calibration as in Clark et al.,[1] reproduced here with minor modifications.

We specified a system of ordinary differential equations defining the derivatives with respect to time of a set of state variables, to simulate the country-specific tuberculosis epidemic between 1900 and 2035. We initialised the simulation by distributing the population between the eight tuberculosis natural history states using a fitted parameter representing the proportion of the population uninfected at the start of the simulation. For each year of the simulation (1900–2035), our models are designed to exactly match the age and country-specific UN population estimates and projections.

Broadly, the modelling approach was as follows:

1. Construct a mechanistic model
2. Calibrate the model by identifying areas of the input parameter space where the output of the mechanistic model was consistent with the historical epidemiologic data
3. Use the calibrated model to simulate and predict future tuberculosis epidemiology and model interventions

In the context of this analysis, step 1 was achieved by creating the compartment differential equation model as specified in Section 1. For step 2, we independently calibrated a model by identifying areas of the parameter space that made the output of the model match the corresponding calibration targets (Table S4 below). The model was fitted to the calibration targets using history matching with emulation, a method that allowed us to explore high-dimensional parameter spaces efficiently and robustly.[28–31] History matching progressed as a series of iterations, called waves, where implausible areas of the parameter space, i.e., areas that were unable to give a match between the model output (e.g., the predicted incidence rate by the model) and the empirical data (e.g., the incidence rate calibration target from WHO data), were found and discarded. In order to identify implausible parameter sets, emulators, which are statistical approximations of model outputs that are built using a modest number of model runs, were used. Emulators provide an estimate of the value of the model at any parameter set of interest, with the advantage that they are orders of magnitude faster than the model.

Table S4 TB model calibration targets for India

Calibration Targets	Year	Age (years)	Estimate	Lower	Upper
Tuberculosis incidence rate (per 100,000 population/year)	2000	All	289	149	473
	2020	All	188	129	257
		0–14	91	56	126
		≥15	224	138	310
Tuberculosis mortality rate (per 100,000 population/year)	2000	All	67	57	79
	2020	All	37	34	40
Tuberculosis case notification rate (per 100,000 population/year)	2000	All	177	142	212
	2020	All	136	109	163
		0–14	33	26	40
		≥15	173	138	208
Active tuberculosis prevalence (per 100,000 population)	2015[32]	All	315	210	529
	2021[33]	All	312	218	406
	2021[33]	≥15	394	276	512
Tuberculosis infection prevalence proportion	2021[33]	All	0.314	0.114	0.514
Subclinical tuberculosis prevalence ratio	2020[34]	All	0.504	0.361	0.797

History matching with emulation, implemented through the hmer package in R,[35,36] considerably reduced the size of the parameter space to investigate. Rejection sampling was then performed on the reduced space to identify at least 1000 parameter sets that matched all targets. If we were unable to find at least 1000 fully fitted parameter sets using history matching with emulation, we subsequently used an Approximate Bayesian Computation using Markov Chain Monte Carlo method (ABC-MCMC). ABC-MCMC was conducted using the easyABC package in R, modified by Sebastian Funk, Gwenan Knight, and the Tuberculosis Modelling group at LSHTM for adaptive sampling and to accept seeded parameter values.[37,38] We used parameter sets with the maximum number of targets fitted using history matching with emulation as starting seeds for multiple MCMC chains, with the ABC-MCMC algorithm continuously adapting using the last 1000 points, a burn in of 1000 samples, and the noise factor set to 0.0001.

Once we had obtained 1000 parameter sets that produced output consistent with the calibration targets, we used those parameter sets with the mechanistic model to simulate the future (step 3). We assumed the

quality and coverage of current TB interventions would remain constant after 2019 until the end of the simulations in 2040. Simulating until 2040 was a stylised approach that provided insight into the short-term implications, as well as a better understanding of how the global system could be reconfigured over a longer time period, and the effect of that on TB.

We validated our model outputs by comparing to estimates of the population attributable fraction (PAF) for undernutrition. The PAF was calculated as:

$$\frac{I_{thin} - I_{not\ thin}}{I_{thin}} \times \frac{D_{thin}}{D}$$

Where I_{thin} represented the incidence of disease in those with moderate to severe thinness and mild thinness, $I_{not\ thin}$ represented the incidence of disease in those with normal or overweight BMI, D_{thin} was the number of TB cases in those with moderate to severe thinness and mild thinness, and D was the number of TB cases overall.

We also used self-reported TB status by BMI strata from the India 2015–2016 Demographic and Health Survey as a validation.[21]

2. Climate and food systems model

2.1 LandSyMM

The Land System Modular Model (LandSyMM, https://landsymm.earth), is a state of the art global land use model that integrates a dynamic global vegetation model (LPJ-GUESS)[39] with a land system model (PLUM)[40] and a Modified, Implicit, Directly Additive Demand System (MAIDADS).[41] LandSyMM combines spatially-explicit, biophysically-derived yield responses with socio-economic scenario data to project future demand, land use, and land management inputs. Here, LandSyMM used climate input data from the fifth Coupled Model Intercomparison Project (CMIP5)[42] for the IPSL-CM5A-MR climate model.[43] LandSyMM improves upon existing integrated assessment models (IAMs) by (a) modelling crop yield responses in a more detailed manner at a finer grain, and (b) calculating commodity demand endogenously and therefore, unlike most land use models, demand for commodities responds dynamically to changing commodity prices.

The MAIDADS system uses per-capita income levels, food prices and price elasticities to estimate subsistence and discretionary consumption levels and captures nonlinear relationships between food demand and income. As incomes rise, consumption shifts away from staple foods (cereals, oil crops and pulses) towards greater consumption of meat and fruit and vegetables. Conversely, as prices increase, overall consumption decreases and shifts away from ‘luxury’ goods such as meat, fruit and vegetables back towards staple crops. If subsistence levels of consumption are too expensive for a country, then demand for food products is calculated by scaling desired subsistence consumption by the ratio between income available for food expenditure and the desired subsistence consumption.

Increasing demand for commodities is met by in-country expansion or intensification of crop production or by imports from the global market. Excess commodity production in a country is exported to the global market. Bilateral trading is not currently modelled in PLUM. LandSyMM reflects reality in agricultural markets where supply adjusts slowly and therefore will not always meet demand in a particular year. The global market is not constrained to be in equilibrium, instead allowing over- or under-supply of commodities buffered through global stocks. Global market prices are adjusted on an annual basis based on the net balance of imports and exports. For example, oversupply of a commodity on the global market decreases the price as stocks rise, this reduces the benefits from its export and reduces the cost of importing it. This model was used to project future estimates of BMI distribution.

2.2 Population weight distributions

We calculated the proportion of the population that was underweight (BMI < 18.5 kg/m^2), normal weight (BMI 18.5–25 kg/m^2), overweight (BMI 25–30 kg/m^2) or obese (BMI > 30.0 kg/m^2) globally and in India for a given year by estimating the mean BMI to use as input in a log normal distribution.[44]

We estimated the mean BMI of a country's population using the following relationship:

$$\begin{aligned}\text{meanBMI}_{c,t} = 11.9 + \ \text{coef}_c + \ \text{kcalPc}_{c,t} \times 0.0037 + \ \text{kcalPc}^2_{c,t} \times -0.0000002 \\ + \ \text{percAP}_{c,t} \times 0.2276 + \ \text{percAP}^2_{c,t} \times 0.0046 + \epsilon\end{aligned} \quad (1)$$

Where coef_c was a country fixed effect, kcalPc was the average calorie consumption per person per day in a country, percAP was the percentage of daily calories consumed in the form of animal products in a country, and ϵ represented the error term. The relationship in *(1)* was estimated by regressing food consumption data from The Food and Agriculture Organization Corporate Statistical Database with WHO estimates of mean BMI for the years 2000–2017 (R^2 = 0.87).

We used the estimated mean BMI of a country to calculate the different population weight proportions for a given time step according to a log normal distribution with a mean:

$$\text{mean}_\text{t} \ = log(\text{meanBMI}_{\text{c,t}}) - \frac{\sigma_\text{c}^2}{2} \quad (2)$$

and standard deviation:

$$\text{sd} = \sigma_c \quad (3)$$

Where σ_c was constant over time and calculated by fitting a log-normal distribution to WHO estimates of mean BMI and the prevalence of underweight, overweight and obesity in 2010 using a cross-entropy method. The cross-entropy approach estimates the parameters of the log-normal distribution by comparing two probability distributions and minimising the Kullback-Leibler Divergence. We estimated moderate to severe thinness (as a proportion of all thinness) by assuming that trends in this identified above from the India Health and Demographic Survey continued into the future.

3. Economic analysis methods

We used the same economic analysis methods as in Clark et al., 2023, reproduced here with minor modifications.[1] Before undertaking this work, we established an economic analysis plan to ensure we had incorporated all necessary information and planned to report on all key outcomes, to outline the methods used in this work. This is summarised below.

3.1 Calculation of disability-adjusted life years

We calculated the difference in total disability-adjusted life years (DALYs) from nutritional intervention introduction to 2035 for each scenario compared to the no-nutritional intervention baseline. We used the disability weight for tuberculosis disease from the Global Burden of Disease 2019 study,[45] and age-specific life expectancy estimates for India overall from the United Nations Development Programme.[46] We did not incorporate any disability weights for different BMI categories. To incorporate parameter uncertainty in years lost due to disability weight estimates, we made 1000 draws from disability weight uncertainty ranges.

3.2 Tuberculosis-related and intervention costs

We estimated health system unit costs, patient costs and productivity losses based on a scoping review of published literature. For the tuberculosis programme, we obtained unit costs for drug-susceptible and drug-resistant tuberculosis diagnostic and treatment costs, which are provided in Table S5. Uncertainty in tuberculosis-related cost estimates was characterised through gamma distributions around plausible unit cost estimates in a probabilistic sensitivity analysis.

Intervention costs per patient and per household contact per month were provided in Bhargava et al.[47] Assuming an average of six months for treatment, we assumed a total cost per patient receiving RATIONS was USD$92.02 and per household contact was USD$33.23.

Costs from the health system perspective included intervention costs (nutritional support per patient or per household contact for the duration of treatment), the cost of testing and diagnosis for drug-susceptible and drug-resistant cases, and the cost of treatment for drug-susceptible and drug-resistant cases. In addition to costs from the health-system perspective, costs from the societal perspective included non-medical patient costs (including transportation) and indirect patient costs for drug-susceptible and drug-resistant cases.

Table S5 Tuberculosis testing, diagnostic, and intervention related cost inputs

Unit Cost	Estimate in USD$	Source
Unit cost of testing/diagnosis for drug-susceptible TB per case diagnosed	22.45 (18.37–26.53)	48
Unit cost of testing/diagnosis for drug-resistant TB per case diagnosed	24.36 (5.04–117.81)	49
Unit cost of treatment for drug-susceptible TB per person	317.00 (254.00–374.00)	50
Unit cost of treatment for drug-resistant TB per person	3,891.00 (3,382.00–4,401.00)	51
Non-medical patient cost per drug-susceptible tuberculosis disease episode (including transportation) per person	51.25 (22.12–76.94)	52,53
Indirect patient cost per drug-susceptible tuberculosis disease episode (time spent on treatment and transport × wage) per person	117.01 (24.04–460.24)	53,54
Non-medical patient cost per drug-resistant tuberculosis disease episode (including transportation) per person	143.49 (61.95–215.42)	52,53
Indirect patient cost per drug-resistant tuberculosis disease episode (time spent on treatment and transport × wage) per person	327.63 (67.30–1,288.66)	53,54
Intervention cost per person receiving TB treatment	92.02	47
Intervention cost per household contact	33.23	47

3.3 Cost-effectiveness analysis and willingness-to-pay thresholds

We calculated the incremental cost effectiveness ratio (ICER) as the ratio between the incremental benefit, in DALYs averted, and the incremental cost, in USD, for each run across intervention and baseline scenario. Both costs and benefits were discounted to 2023 (when the intervention began) at 3% per year, per guidelines.[55] We measured cost-effectiveness by 2035 against three India specific cost thresholds: 1 x gross domestic product (GDP) per-capita (US$2,411), and two country-level opportunity cost thresholds defined by Ochalek et al [the upper (US$555), and lower (US$410) bounds].[56]

SUPPLEMENTARY RESULTS

4. Epidemiological trends in the *No Intervention* baseline scenario

Trends in tuberculosis epidemiology from 1980–2040 for the *No Intervention* baseline scenario are shown in Figure S3 below.

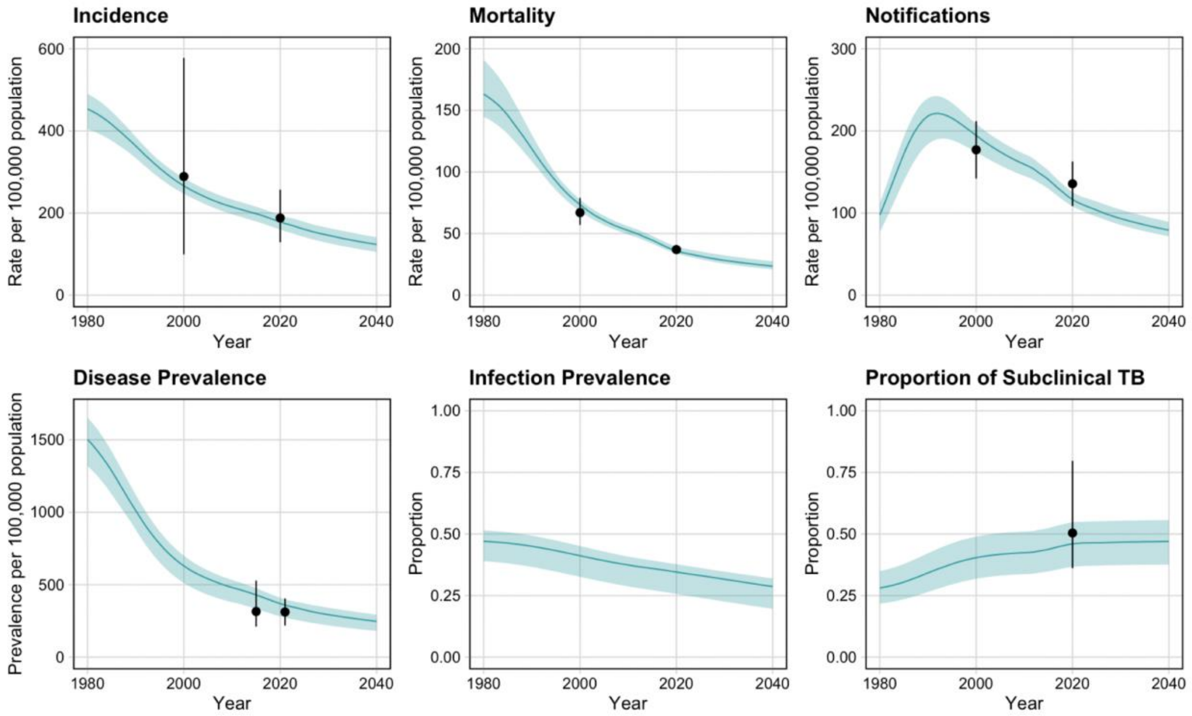

Figure S3 Model simulated baseline trends in epidemiology

Trends in BMI in the No Intervention baseline scenario

In the *No Intervention* baseline scenario, 12.5% (95% uncertainty interval = 11.8, 13.4) and 8.5% (8.1, 9.1) of the population in 2022 overall was predicted to have mild and moderate to severe thinness respectively, compared to 18.1% (17.3, 19.2) and 17.9% (17.0, 18.7) among people with TB. In 2035, the proportion of the population with mild thinness and moderate to severe thinness was predicted to decrease to 10.0% (9.5, 10.6) and 6.3% (6.0, 6.7) respectively, with corresponding declines in the proportion of mild and moderate to severe thinness among people with TB (16% (15.3, 16.8) and 14.8% (14.2, 15.5) respectively). In the *No Intervention* baseline scenario scenario in 2022, 16.4% (15.2, 17.5) of the population was predicted to have an overweight BMI, compared to 5.1% (4.1, 6) among people with TB. The proportion of the population with an overweight BMI was predicted to increase in 2035, with 21.8% (20.8, 22.7) of the overall population, and 6.8% (6.3, 7.3) among people with TB.

Validation with PAF and self-reported TB incidence by BMI strata

Estimates of the PAF for undernutrition for TB are provided in Table S6, and comparisons between the model output TB incidence by BMI strata for all ages and adults and the self-reported TB incidence by BMI strata from the India 2015–2016 Demographic and Health Survey are in Table S7. The model predicted PAF was 18.4% (95% uncertainty interval = 17.8, 19.1), which falls on the lower end of the range of the revised and updated estimate from Bhargava et al., 2022 (45.2% [17.0, 71.0]). Similarly, the model predicted a lower proportion of TB in the lower BMI strata compared to estimates (Table S7). Whereas the self-reported incidence of TB (which may differ from actual incidence of TB due to access to diagnosis and care, recall bias or other factors) in those with moderate to severe thinness was 1,093 (956, 1 230) per 100,000 population, the model predicted incidence for adults was almost 2.5 times lower.

As noted in section 1.2, there was limited data available to capture the relationship between low BMI and TB. The model was calibrated to reported estimates of incidence overall, using risk ratios from Table S3, where the maximum for the increase in progression to TB disease for moderate to severe thinness is less than 2 times the rate for those with normal BMI. The low rate ratios may have resulted in an underestimation of the amount of TB that occurred in the two low BMI strata. Additionally, individuals are assumed to mix randomly in the model, which may not be the case in reality, and it is likely that assortative mixing would contribute to increasing the burden of TB in lower BMI strata.

Table S6 Estimates of the population attributable fraction of undernutrition for India

**Study and measure**	**PAF for India (95% CI)**
WHO estimates, 2023	26.4% (22.3, 30.8)
Bhargava., 2022<br>(Using prevalence of undernourishment and RR = 3.2)	24.8%
Bhargava., 2022<br>(Using prevalence of undernutrition and original RR = 3.2	34.2% (28.6, 39.3)
Bhargava., 2022<br>(Using prevalence of undernutrition and revised RR = 4.49 (2.28, 8.86))	45.2% (17.0, 71.0)
Model predicted PAF	18.4% (17.8, 19.1)

Table S7 Self-reported TB incidence by BMI strata

BMI strata	BMI interval	Self-reported TB in 2015 per 100,000 [95% CI]	Model TB in 2015 per 100,000 for all ages [95% UI]	Model TB in 2015 per 100,000 for adults [95% UI]
Moderate to severe thinness	<17.0 kg/m^2	1 093<br>(956, 1 230)	375.6<br>(342.8, 406.2)	441.9<br>(399.9, 484.8)
Mild thinness	17.0–18.4 kg/m^2	431<br>(367, 496)	273.7<br>(254.6, 293.9)	325.3<br>(299.6, 355.4)
Normal BMI	18.5–24.9 kg/m^2	234<br>(209, 260)	185.3<br>(172.3, 199)	222<br>(205.7, 241.8)
Overweight to obese BMI	≥25.0 kg/m^2	145<br>(11, 181)	53.3<br>(47.7, 61.2)	55.5<br>(49.4, 64.1)

5. Intervention scenario results

Supplementary health, cost, and cost-effectiveness results for all scenarios, coverages, and durations of protection are provided on github.

Our results demonstrate that the majority of intervention benefit in terms of TB disease averted is a result of reduced TB incidence in household contacts of TB patients; indeed an improvement in treatment outcomes for TB patients paradoxically leads to a slight increase in incidence, as these individuals may be more likely to experience recurrent TB. This may no longer be the cases if the nutritional intervention is shown to decrease recurrence rates, for which undernutrition is a risk factor. In contrast, intervention benefit in terms of TB deaths averted is a result of both reduced TB incidence in household contacts (who are therefore less likely to die of TB) and improved outcomes in TB patients. This demonstrates the importance of nutritional support for TB patients as well as their household contacts in reducing TB deaths.

The duration of protection we assumed in our intervention did not affect our results significantly. As improvements in BMI were not important drivers in the intervention, the rate at which these improvements waned did not affect results. However, due to the structure of our model (which does not explicitly model households) we were unable to vary the duration of protection against progression to TB disease in household contacts of people with TB. The approach assumes a given number of people who were at risk of progressing to TB disease do not in fact do so, and is time-independent (and therefore independent of the duration of protection). Given that this accounted for the majority of reduction in TB incidence, and represents a group of individuals likely at higher long-term risk of TB disease than the general population, these results should be interpreted with caution.

5.1 Health impact results

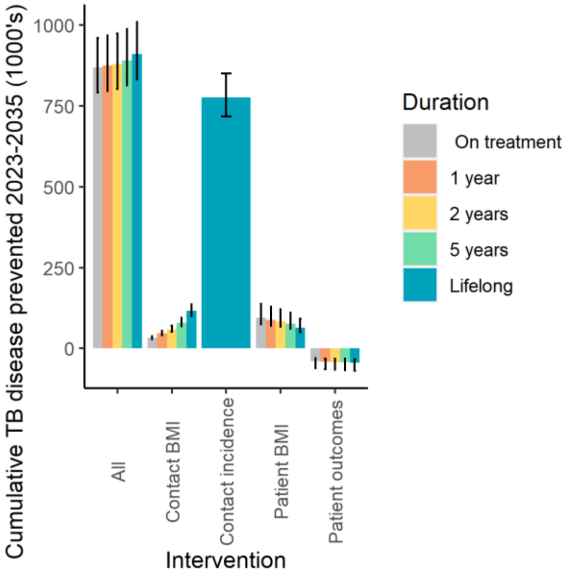

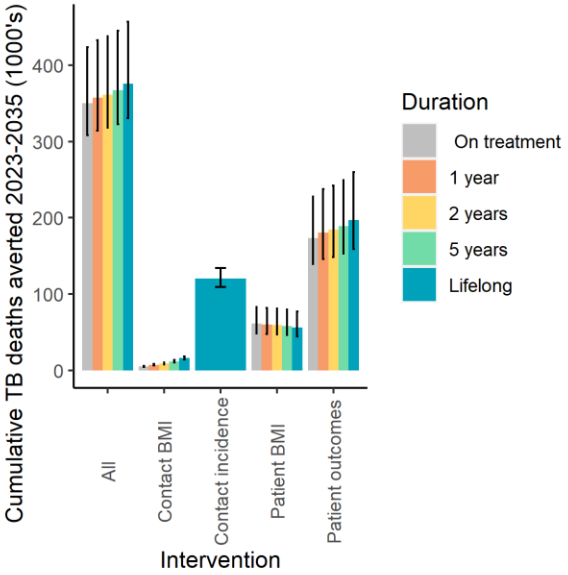

Figure S4 Cumulative number of people prevented from (a) developing tuberculosis disease or (b) dying of tuberculosis due to a nutritional support intervention with 50% coverage of adults receiving tuberculosis treatment in India between 2023 and 2035, assuming varying durations of protection and considering separate mechanisms of action; all mechanisms together, or separately whether the intervention improved the body mass index of household contacts, reduced the incidence of tuberculosis in household contacts, improved the body mass index of people with tuberculosis, or reduced tuberculosis mortality in people with tuberculosis. TB=tuberculosis, BMI=body mass index

Varying the intervention effect by BMI status saw a decreased intervention effect when varying treatment outcomes (assuming a risk ratio of 0.62 for BMI < 17.0 kg/m^2, 0.72 for BMI 17.0-18.4 kg/m^2, 0.80 for BMI 18.5-24.9 kg/m^2 and 0.98 for BMI ≥ 25.0 kg/m^2, based on the same adjusted hazard ratio and the proportion in each group who experienced a weight gain ≥5% at 2 months), and an increased effect when varying the prevention of incident TB. This is a result of the differing BMI distribution in the model population compared to the trial, with an increase in mean BMI. As a result, while improved outcomes in those with low BMI do have a larger effect in a population at greater risk of poor treatment outcomes, this is not sufficient to make up for the reduced size of the population affected. Similarly, while there was a reduced effect of the intervention in household contacts with low BMI, due to the larger population in our model with a normal BMI the intervention effect size increased overall.

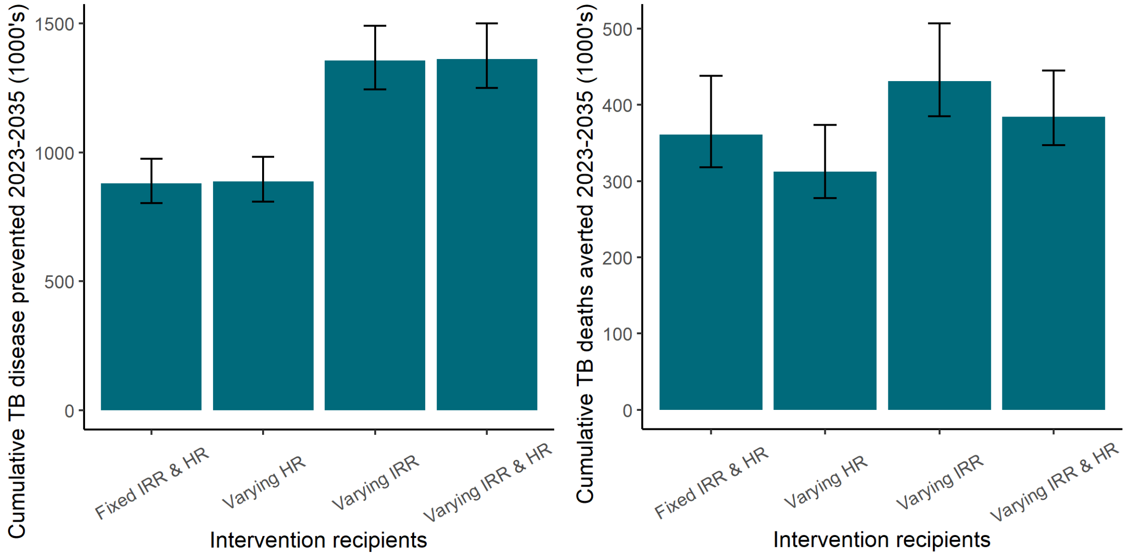

Figure S5 Cumulative TB disease episodes and deaths prevented with hazard ratio and incident rate ratio varying by BMI status. Primary analysis results with the same ratios across different BMI strata (Fixed IRR & HR) are compared to scenarios where the hazard of death (HR) and incident rate ratio (IRR) are varied separately and together.

5.2 Cost-effectiveness results

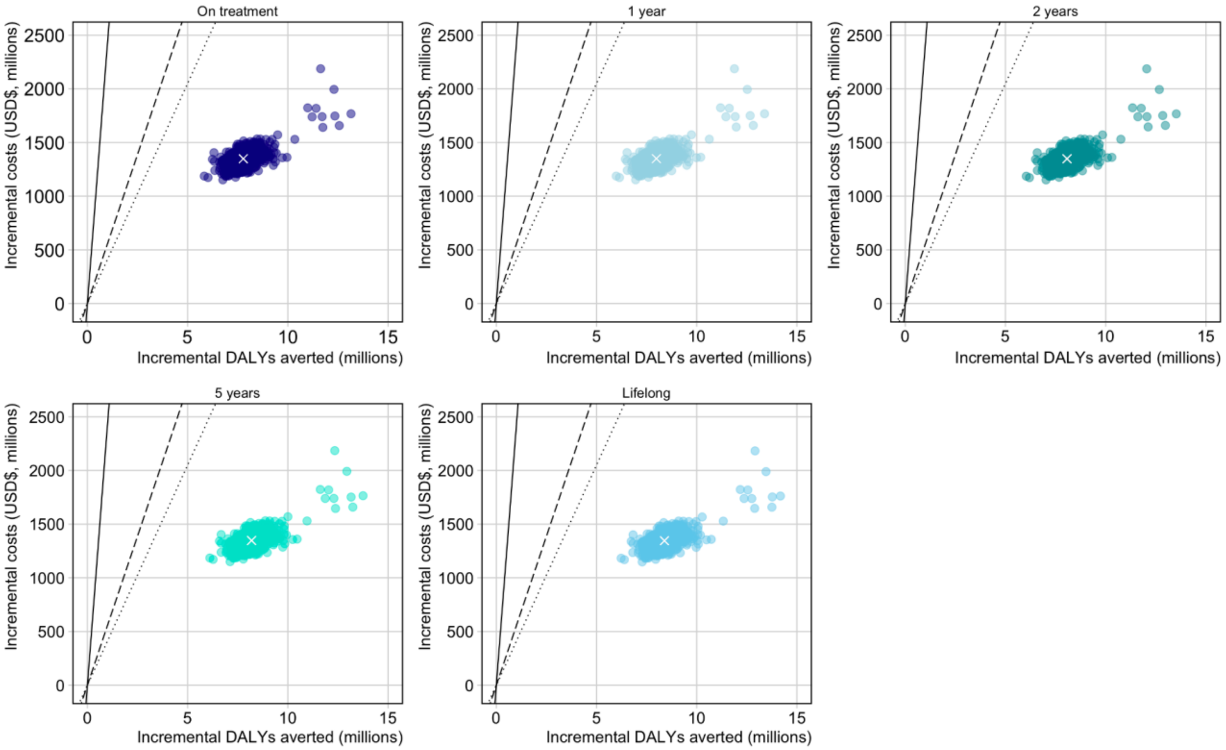

Figure S6 Cost-effectiveness planes for scenarios with RATIONS delivered to the whole household compared to *No Intervention* with 50% coverage by varying durations of protection. Each point represents the costs and benefits for an individual model run, and the white cross represents the mean costs and benefits for the 1000 runs.

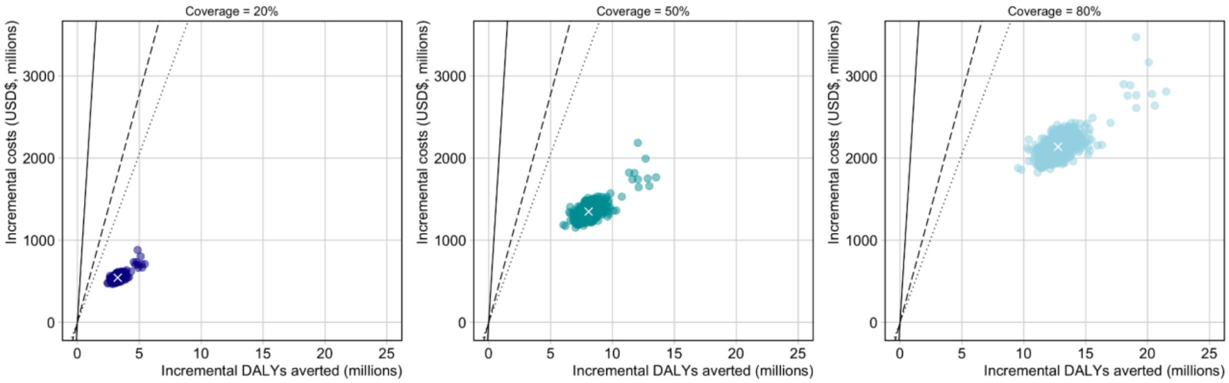

Figure S7 Cost-effectiveness planes for scenarios with RATIONS delivered to the whole household compared to *No Intervention* with 2 years duration of protections and varying coverage. Each point represents the costs and benefits for an individual model run, and the white cross represents the mean costs and benefits for the 1000 runs.

5.3 Budget Impact results

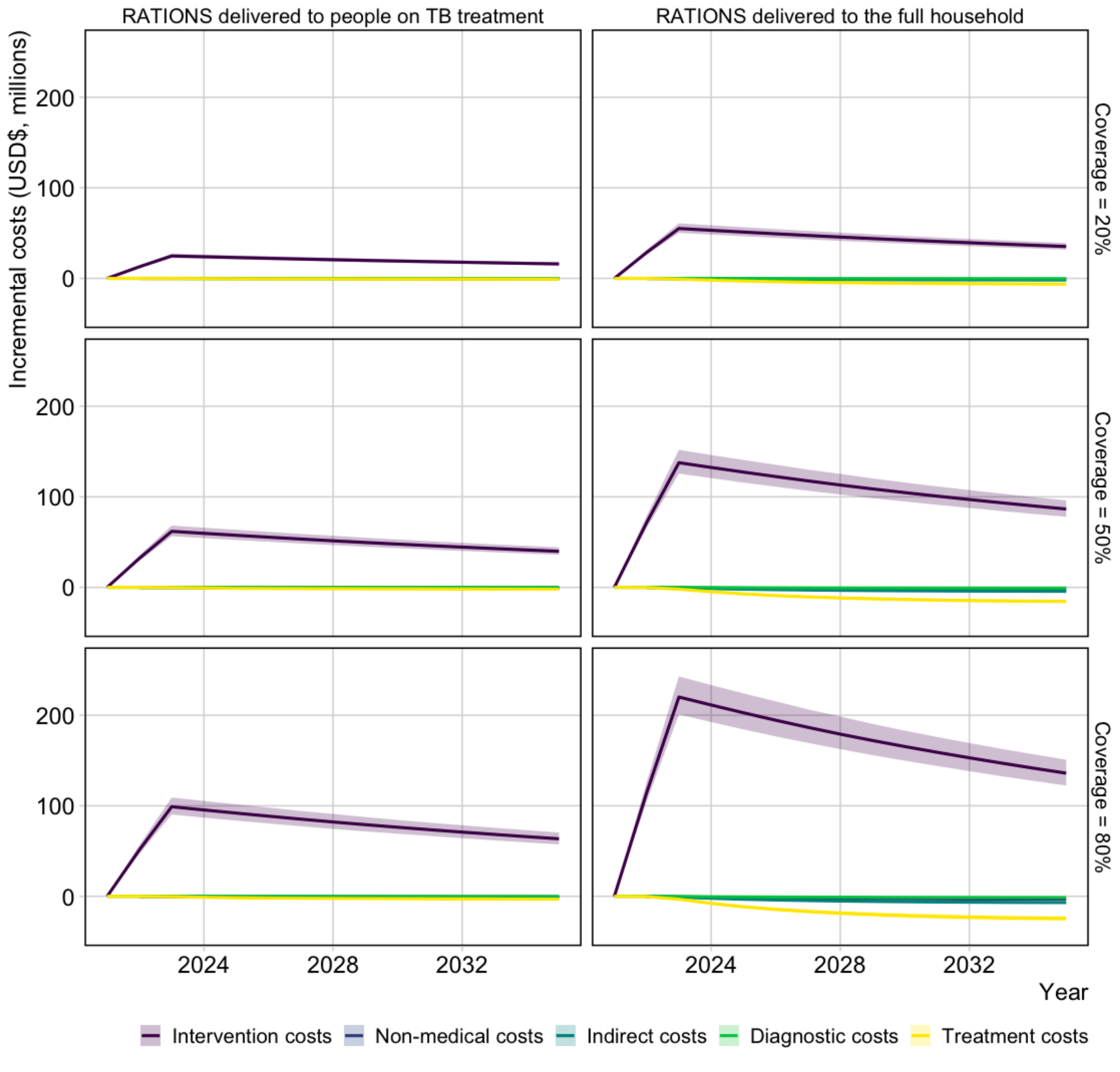

Figure S8 Incremental costs compared to *No Intervention* for delivering RATIONS to people on TB treatment only or to the full household for 20%, 50%, and 80% coverage assuming 2 years protection.

REFERENCES

1 Clark RA, Weerasuriya CK, Portnoy A, *et al.* New tuberculosis vaccines in India: Modelling the potential health and economic impacts of adolescent/adult vaccination with M72/AS01E and BCG-revaccination. *BMC Med* 2023; **21**. DOI:https://doi.org/10.1186/s12916-023-02992-7.

2 Rajagopalan S. Tuberculosis and aging: a global health problem. *Clin Infect Dis Off Publ Infect Dis Soc Am* 2001; **33**: 1034–9.

3 United Nations, Department of Economic and Social Affairs, Population Division. World Population Projections [2019 Revision]. 2019. https://population.un.org/wpp/Download/Standard/Population/ (accessed Nov 2, 2022).

4 Tiemersma EW, Werf MJ van der, Borgdorff MW, Williams BG, Nagelkerke NJD. Natural History of Tuberculosis: Duration and Fatality of Untreated Pulmonary Tuberculosis in HIV Negative Patients: A Systematic Review. *PLOS ONE* 2011; **6**: e17601.

5 Quaife M, Houben RMGJ, Allwood B, *et al.* Post-tuberculosis mortality and morbidity: valuing the hidden epidemic. *Lancet Respir Med* 2020; **8**: 332–3.

6 Prem K, Zandvoort K van, Klepac P, *et al.* Projecting contact matrices in 177 geographical regions: An update and comparison with empirical data for the COVID-19 era. *PLOS Comput Biol* 2021; **17**: e1009098.

7 World Health Organization. WHO TB burden estimates. CSV Files Download. 2022. https://www.who.int/tb/country/data/download/en/ (accessed Nov 2, 2022).

8 World Health Organization. Case Notifications. CSV Files Download. 2022. https://www.who.int/tb/country/data/download/en/ (accessed Nov 2, 2022).

9 Emery J. Subclinical TB disease: lessons and questions from field to lab. 2020.

10 Emery JC, Richards AS, Dale KD, *et al.* Self-clearance of *Mycobacterium tuberculosis* infection: implications for lifetime risk and population at-risk of tuberculosis disease. *Proc R Soc B Biol Sci* 2021; **288**: 20201635.

11 Abu-Raddad L, Sabatelli L, Achterberg JT, *et al.* Epidemiological benefits of more-effective tuberculosis vaccines, drugs, and diagnostics. *Proc Natl Acad Sci U S A* 2009; **106**: 13980–5.

12 Dye C, Williams BG. Eliminating human tuberculosis in the twenty-first century. *J R Soc Interface* 2008; **5**: 653–62.

13 Marx FM, Dunbar R, Enarson DA, *et al.* The Temporal Dynamics of Relapse and Reinfection Tuberculosis After Successful Treatment: A Retrospective Cohort Study. *Clin Infect Dis* 2014; **58**: 1676–83.

14 Gomes MGM, Franco AO, Gomes MC, Medley GF. The reinfection threshold promotes variability in tuberculosis epidemiology and vaccine efficacy. *Proc R Soc B Biol Sci* 2004; **271**: 617–23.

15 Dangisso MH, Woldesemayat EM, Datiko DG, Lindtjørn B. Long-term outcome of smear-positive tuberculosis patients after initiation and completion of treatment: A ten-year retrospective cohort study. *PloS One* 2018; **13**: e0193396.

16 Sutherland I, Svandová E, Radhakrishna S. The development of clinical tuberculosis following infection with tubercle bacilli. 1. A theoretical model for the development of clinical tuberculosis following infection, linking from data on the risk of tuberculous infection and the incidence of clinical tuberculosis in the Netherlands. *Tubercle* 1982; **63**: 255–68.

17 Vynnycky E, Fine PE. The natural history of tuberculosis: the implications of age-dependent risks of disease and the role of reinfection. *Epidemiol Infect* 1997; **119**: 183–201.

18 Gabriela M. Gomes M, Rodrigues P, Hilker FM, *et al.* Implications of partial immunity on the prospects for tuberculosis control by post-exposure interventions. *J Theor Biol* 2007; **248**: 608–17.

19 Global Health Observatory. https://www.who.int/data/gho (accessed Dec 19, 2023).

20 International Institute for Population Sciences (IIPS) and ICF. National Family Health Survey (NFHS-3), 2005-06. Mumbai, 2007.

21 International Institute for Population Sciences (IIPS) and ICF. National Family Health Survey (NFHS-4), 2015-16. Mumbai, 2017.

22 International Institute for Population Sciences (IIPS) and ICF. National Family Health Survey (NFHS-5), 2019-21. Mumbai, 2022.
23 Lumley T. survey: Analysis of Complex Survey Samples. 2023; published online May 3. https://cran.r-project.org/web/packages/survey/index.html (accessed Feb 5, 2024).
24 Lönnroth K, Williams BG, Cegielski P, Dye C. A consistent log-linear relationship between tuberculosis incidence and body mass index. *Int J Epidemiol* 2010; **39**: 149–55.
25 Cegielski JP, Arab L, Cornoni-Huntley J. Nutritional Risk Factors for Tuberculosis Among Adults in the United States, 1971–1992. *Am J Epidemiol* 2012; **176**: 409–22.
26 Oxlade O, Huang C-C, Murray M. Estimating the Impact of Reducing Under-Nutrition on the Tuberculosis Epidemic in the Central Eastern States of India: A Dynamic Modeling Study. *PLOS ONE* 2015; **10**: e0128187.
27 Sinha P, Ponnuraja C, Gupte N, *et al.* Impact of Undernutrition on Tuberculosis Treatment Outcomes in India: A Multicenter, Prospective, Cohort Analysis. *Clin Infect Dis* 2023; **76**: 1483–91.
28 Andrianakis I, Vernon I, McCreesh N, *et al.* History matching of a complex epidemiological model of human immunodeficiency virus transmission by using variance emulation. *J R Stat Soc Ser C Appl Stat* 2017; **66**: 717–40.
29 Andrianakis I, Vernon IR, McCreesh N, *et al.* Bayesian History Matching of Complex Infectious Disease Models Using Emulation: A Tutorial and a Case Study on HIV in Uganda. *PLOS Comput Biol* 2015; **11**: e1003968.
30 Goldstein M. Bayes Linear Analysis for Complex Physical Systems Modeled by Computer Simulators. In: Dienstfrey AM, Boisvert RF, eds. Uncertainty Quantification in Scientific Computing. Berlin, Heidelberg: Springer Berlin Heidelberg, 2012: 78–94.
31 Williamson D, Goldstein M, Allison L, *et al.* History matching for exploring and reducing climate model parameter space using observations and a large perturbed physics ensemble. *Clim Dyn* 2013; **41**: 1703–29.
32 Pandey S, Chadha VK, Laxminarayan R, Arinaminpathy N. Estimating tuberculosis incidence from primary survey data: a mathematical modeling approach. *Int J Tuberc Lung Dis* 2017; **21**: 366–74.
33 Indian Council of Medical Research (ICMR). National TB prevalence survey India 2019-2021. 2022.
34 Frascella B, Richards AS, Sossen B, *et al.* Subclinical tuberculosis disease - a review and analysis of prevalence surveys to inform definitions, burden, associations and screening methodology. *Clin Infect Dis* 2021; **73**: e830–41.
35 Iskauskas A. hmer: History Matching and Emulation Package. 2022. https://CRAN.R-project.org/package=hmer (accessed Nov 2, 2022).
36 Iskauskas A, Vernon I, Goldstein M, *et al.* Emulation and History Matching using the hmer Package. 2022; published online Sept 12. http://arxiv.org/abs/2209.05265 (accessed Dec 20, 2022).
37 Jabot F, Faure T, Dumoulin N. EasyABC: performing efficient approximate Bayesian computation sampling schemes using R. *Methods Ecol Evol* 2013; **4**: 684–7.
38 Roberts GO, Rosenthal JS. Examples of Adaptive MCMC. *J Comput Graph Stat* 2009; **18**: 349–67.
39 Smith B, Wårlind D, Arneth A, *et al.* Implications of incorporating N cycling and N limitations on primary production in an individual-based dynamic vegetation model. *Biogeosciences* 2014; **11**: 2027–54.
40 Alexander P, Rabin S, Anthoni P, *et al.* Adaptation of global land use and management intensity to changes in climate and atmospheric carbon dioxide. *Glob Change Biol* 2018; **24**: 2791–809.
41 Gouel C, Guimbard H. Nutrition Transition and the Structure of Global Food Demand. *Am J Agric Econ* 2019; **101**: 383–403.
42 Taylor KE, Stouffer RJ, Meehl GA. An Overview of CMIP5 and the Experiment Design. *Bull Am Meteorol Soc* 2012; **93**: 485–98.
43 Dufresne J-L, Foujols M-A, Denvil S, *et al.* Climate change projections using the IPSL-CM5 Earth System Model: from CMIP3 to CMIP5. *Clim Dyn* 2013; **40**: 2123–65.
44 Springmann M, Mason-D'Croz D, Robinson S, *et al.* Global and regional health effects of future food production under climate change: a modelling study. *The Lancet* 2016; **387**: 1937–46.

45 Vos T, Lim SS, Abbafati C, *et al.* Global burden of 369 diseases and injuries in 204 countries and territories, 1990–2019: a systematic analysis for the Global Burden of Disease Study 2019. *The Lancet* 2020; **396**: 1204–22.
46 United Nations Development Programme. Human Development Report: Life Expectancy by Country. United Nations, 2020 https://hdr.undp.org/data-center (accessed June 13, 2022).
47 Bhargava A, Bhargava M, Meher A, *et al.* Nutritional supplementation to prevent tuberculosis incidence in household contacts of patients with pulmonary tuberculosis in India (RATIONS): a field-based, open-label, cluster-randomised, controlled trial. *The Lancet* 2023; **402**: 627–40.
48 Vassall A, Kampen S van, Sohn H, *et al.* Rapid Diagnosis of Tuberculosis with the Xpert MTB/RIF Assay in High Burden Countries: A Cost-Effectiveness Analysis. *PLOS Med* 2011; **8**: e1001120.
49 Muniyandi M, Lavanya J, Karikalan N, *et al.* Estimating TB diagnostic costs incurred under the National Tuberculosis Elimination Programme: a costing study from Tamil Nadu, South India. *Int Health* 2021; **13**: 536–44.
50 Chatterjee S, Toshniwal MN, Bhide P, *et al.* Costs of TB services in India. *Int J Tuberc Lung Dis* 2021; **25**: 1013–8.
51 Gotham D, Fortunak J, Pozniak A, *et al.* Estimated generic prices for novel treatments for drug-resistant tuberculosis. *J Antimicrob Chemother* 2017; **72**: 1243–52.
52 Sinha P, Carwile M, Bhargava A, *et al.* How much do Indians pay for tuberculosis treatment? A cost analysis. *Public Health Action* 2020; **10**: 110–7.
53 Sarin R, Vohra V, Singla N, Thomas B, Krishnan R, Muniyandi M. Identifying costs contributing to catastrophic expenditure among TB patients registered under RNTCP in Delhi metro city in India. *Indian J Tuberc* 2019; **66**: 150–7.
54 Chandra A, Kumar R, Kant S, Parthasarathy R, Krishnan A. Direct and indirect patient costs of tuberculosis care in India. *Trop Med Int Health TM IH* 2020; **25**: 803–12.
55 Wilkinson T, Sculpher MJ, Claxton K, *et al.* The International Decision Support Initiative Reference Case for Economic Evaluation: An Aid to Thought. *Value Health* 2016; **19**: 921–8.
56 Ochalek J, Lomas J, Claxton K. Estimating health opportunity costs in low-income and middle-income countries: a novel approach and evidence from cross-country data. *BMJ Glob Health* 2018; **3**: e000964.
