# Supplementary material for: Estimating the epidemiological and economic impact of providing nutritional care for tuberculosis-affected households across India: a modelling study
Source: Lancet Glob Health. 2025 Jan 14;13(3):e488–96. doi: 10.1016/S2214-109X(24)00505-9 (PMC11865009; doi:10.1016/S2214-109X(24)00505-9)
Supplement: Equitable Partnership Declaration [file mmc4.pdf]

THE LANCET
Global Health

Supplementary appendix 4

This Equitable Partnership Declaration (EPD) was submitted by the authors, and we reproduce it as supplied. It has not been peer reviewed. *The Lancet*'s editorial processes have not been applied to the EPD.

Supplement to: McQuaid CF, Clark RA, White RG, et al. Estimating the epidemiological and economic impact of providing nutritional care for tuberculosis-affected households across India: a modelling study. *Lancet Glob Health* 2025; published online Jan 14. https://doi.org/10.1016/S2214-109X(24)00505-9.

Equitable Partnership Declaration questions

This Equitable Partnership Declaration is a statement being published online alongside papers at *The Lancet Global Health*, as a separate appendix, to allow researchers to describe how their work engages with researchers, communities, and environments in the countries of study. This is part of our broader goal to decolonise global health, handing control and leadership of research to academics and clinicians who are based in the regions of study, and to affected communities.

Please answer all questions with as much detail as possible, noting that all included information will be published open-access and it will be freely available online to all who wish to read it. If a question does not apply to your study, please state "Not applicable".

The format of and questions in this statement are currently in a pilot phase. Please email Dr Kate McIntosh (Kate.McIntosh@lancet.com; deputy editor) with any feedback, particularly if you find any questions unclear.

Researcher considerations

1. Please detail the involvement that researchers who are based in the region(s) of study had during a) study design; b) clinical study processes, such as processing blood samples, prescribing medication, or patient recruitment; c) data interpretation; and d) manuscript preparation, commenting on all aspects. If they were not involved in any of these aspects, please explain why.

   This question is intended for international partnerships; if all your authors are based in the area of study, this question is not applicable.

   This should include a thorough description of their leadership role(s) in the study. Are local researchers named in the author list or the acknowledgements, or are they not mentioned at all (and, if not, why)? Please also describe the involvement of early career researchers based in the location of the study. Some of this information might be repeated from the Contributors section in the manuscript. Note: we adhere to ICMJE authorship criteria when deciding who should be named on a paper.

**a) Study design:**<br>Principle investigators of the trial on which this modelling study is based, who are themselves based in the region of the study, were involved in design of the study through open discussion of the modelling approaches taken, and are present in the author list.
**b) Clinical study processes: Not applicable**
**c) Data interpretation:**<br>The same investigators were involved in iterative interpretation of the results, which led to revisions and updates.
**d) Manuscript preparation:**<br>The same investigators provided input to the manuscript drafts, including in particular to the background and the discussion and interpretation of results.

2. Were the data used in your study collected by authors named on the paper, or have they been extracted from a source such as a national survey? ie, is this a secondary analysis of data that were not collected by the authors of this paper. If the authors of this paper were not involved in data collection, how were data interpreted with sufficient contextual knowledge?

   The Lancet Global Health *believe contextual understanding is crucial for informed data analysis and interpretation.*

This is a modelling study which did use in part data published elsewhere. Data for the trial (published elsewhere) where interpreted through discussion with the principle investigators of the trial (present on the author list).

3. How was funding used to remunerate and enhance the skills of researchers and institutions based in the area(s) of study? And how was funding used to improve research infrastructure in the area of study?

   Potentially effective investments into long-term skills and opportunities within institutions could include training or mentorship in analytical techniques and manuscript writing, opportunities to lead all or specific aspects of the study, financial remuneration rather than requiring volunteers, and other professional development and educational opportunities.

   Improvements to research infrastructure could be funding of extended trial designs (such as platform trials) and use of master protocols to enable these designs, establishment of long-term contracts for research staff, building research facilities, and local control of funding allocation.

**Skills:**<br>This study was unfunded. However, the trial principle investigators (present on the author list) did enrol on a modelling course run by the selection of other authors on the study, which provided an opportunity to develop their modelling skills and understanding.
**Research infrastructure:**<br>This study was unfunded.

4. How did you safeguard the researchers who implemented the study?

Please describe how you guaranteed safe working conditions for study staff, including provision of appropriate personal protective equipment, protection from violence, and prevention of overworking.

Not applicable

Benefits to the communities and regions of study

5. How does the study address the research and policy priorities of its location?

How were the local priorities determined and then used to inform the research question? Who decided which priorities to take forward? Which elements of the study address those priorities?

The study was based on a clinical trial conducted in the region, which has generated significant local interest in the National TB Programme and elsewhere in both the wider benefit and cost if such an intervention were to be widely implemented. This modelling addressed these priorities.

6. How will research products be shared in the community of study?

For instance, will you be providing written or oral layperson summaries for non-academic information sharing? Will study data be made available to institutions in the region(s) of study? The Lancet Global Health *encourages authors to translate the summary (abstract) into relevant languages after paper editing; do you intend to translate your summary?*

The manuscript abstract will be translated into two local languages. All study data will be made available online.

7. How were individuals, communities, and environments protected from harm?

a) *How did you ensure that sensitive patient data was handled safely and respectfully? Was there any potential for stigma or discrimination against participants arising from any of the procedures or outcomes of the study?*

Not applicable

b) *Might any of the tests be experienced as invasive or culturally insensitive?*

Not applicable

c) *How did you determine that work was sensitive to traditions, restrictions, and considerations of all cultural and religious groups in the study population?*

Not applicable

d) *Were biowaste and radioactive waste disposed of in accordance with local laws?*

Not applicable

e) *Were any structures built that would have impacted members of the community or the environment (such as handwashing facilities in a public space)? If so, how did you ensure that you had appropriate community buy-in?*

Not applicable

f) *How might the study have impacted existing health-care resources (such as staff workloads, use of equipment that is typically employed elsewhere, or reallocation of public funds)?*

Not applicable

8. Finally, please provide the title (eg, Dr/Prof, Mr/Mrs/Ms/Mx), name, and email address of an author who can be contacted about this statement. This can be the corresponding author.

**Name:** Dr Finn McQuaid<br>**Email:** finn.mcquaid@lshtm.ac.uk
